# Micro-food web complexity and stability drive differences in soil multifunctionality of subtropical karst plantations in southwest China

**DOI:** 10.3389/fmicb.2026.1845498

**Published:** 2026-07-10

**Authors:** Yalong Kang, Canfeng Li, Linjun Shen, Yong Huang

**Affiliations:** 1 International Joint Laboratory for Resource Utilization of Agricultural Solid Waste in Yunnan Province, College of Resources and Environmental Science, Yunnan Agricultural University, Kunming, China; 2 Kunming Natural Resources Comprehensive Survey Center of China Geological Survey/Technology Innovation Center for Natural Ecosystem Carbon Sink, Ministry of Natural Resources, Kunming, China; 3 School of Ecology and Environmental Sciences, Yunnan University, Kunming, China; 4 Ministry of Education Key Laboratory for Transboundary Ecosecurity of Southwest China, Kunming, China; 5 School of Ecology and Nature Conservation, Beijing Forestry University, Beijing, China

**Keywords:** hierarchical co-occurrence network, plantations, sensitive taxa, soil multifunctionality index, soil multitrophic microbial diversity, subtropical karst

## Abstract

Afforestation is vital for combating land degradation and restoring ecosystem multifunctions in karst desertification regions. However, the connections between multiple characteristics of microbial communities and soil multifunctionality (SMF) remain poorly understood in the different plantations within subtropical karst regions. Here, using the single function, and averaging method, we calculated the SMF of the two typical types of plantation (i.e., *Pinus armandii* Franch. PAF, and *Alnus nepalensis*, ANP) based on 20 variables associated with soil nutrient provisioning and cycling, soil water retention, microbial growth efficiency, and soil organic matter decomposition. The results showed that the carbon and nitrogen cycling indexes, soil water regulation capacity, and SMF were all significantly greater in the PAF compared to the ANP. The fungal and protistan communities, along with multitrophic microbial β-diversity, positively influenced SMF, while the complexity and stability of bacterial-fungal-protistan networks have a negative impact. Remarkably, changes in the abundance of sensitive taxa within Module 3 adversely impacted the SMF, with sensitive taxa of fungi (e.g., *Trichocladium*) and protists (e.g., *Spongospora*, *Hartmannellidae_X* and *Trinematidae_X*), as opposed to bacteria, playing a crucial role in driving the SMF. Furthermore, carbon and nitrogen sources, exchangeable Mg, and soil water content were determined to be pivotal forcing factors of SMF. Overall, these findings offer a framework for predicting ecosystem functions in subtropical karst forests and valuable insights for managing and conserving fragile ecosystems globally.

## 1 Introduction

At present, global climate change is intensifying and exacerbating, leading to heightened regional climate disparities, which pose significant threats to the diversity, stability, and productivity of terrestrial ecosystems worldwide (Li et al., 2024; Zhou et al., 2024; Frietsch et al., 2026). Karst ecosystems, characterized by rocky outcrops, fissured bedrock, and shallow soils, are particularly fragile and vulnerable to vegetation degradation and rocky desertification (Wang et al., 2019; Cao and Xiong, 2024; Kang et al., 2025; Lei et al., 2025). Compared to natural restoration, afforestation is an effective strategy for rapidly restoring vegetation cover, reducing soil erosion, and enhancing ecological functions in degraded landscapes (Lu et al., 2022). Since 1999, China has implemented large-scale projects to combat rocky desertification, resulting in over 400,000 hm^2^ of new plantations (Wang et al., 2019; Luo et al., 2021; Qiao et al., 2021; Zou et al., 2024). However, plantations in karst regions remain susceptible to degradation due to shallow soils, nutrient limitations, and human disturbances (Zou et al., 2024; Sun et al., 2025). As demonstrated by a plethora of studies, the deterioration of vegetation has been shown to induce a concomitant degradation of numerous ecological functions, including but not limited to carbon sequestration, water holding capacity, nutrient cycling capacity and productivity (Green et al., 2019; Kang et al., 2024). Consequently, sustaining and improving soil ecological functions is essential for successful restoration in karst desertification areas.

Soil ecosystem multifunctionality (SMF) reflects the capacity of soils to support multiple processes such as nutrient cycling, productivity, and biodiversity (Garland et al., 2021; He et al., 2025; Zhou et al., 2026). Soil microorganisms–including bacteria, fungi, and protists–play critical roles in organic matter decomposition, nutrient cycling, and plant productivity, thereby driving SMF (Delgado-Baquerizo et al., 2020; Chen et al., 2022; Wang B. et al., 2025). However, the relationship between soil microbial diversity (α- and β-diversity) and SMF remains inconsistent across studies, showing positive, negative, or nonsignificant correlations (Chen et al., 2023; Che and Jin, 2024; Gong et al., 2024; Kang et al., 2024; Lu et al., 2025; Luo et al., 2025). Thus, understanding microbial mechanisms behind SMF requires looking beyond diversity indices. Soil microorganisms interact through complex trophic networks–forming micro-food webs–via predation, competition, and mutualism (Faust and Raes, 2012). Among these, protists function as key predators that regulate bacterial and fungal communities, promote nutrient release, and thereby modulate SMF (Liao et al., 2024). Recent evidence suggests that the complexity of these networks influences SMF (Wang et al., 2024; Long et al., 2025; Lu et al., 2025), though findings vary across ecosystems and environmental conditions (Wang et al., 2024; Zhu et al., 2024; Chen Z. et al., 2025; Kang et al., 2025; Long et al., 2025; Lu et al., 2025; Luo et al., 2025). Given the high habitat heterogeneity in karst regions (Xiao et al., 2023), more attention should be paid to how microbial diversity and network complexity regulate SMF in plantations, which is key for evaluating restoration outcomes and ecosystem resilience.

Karst ecosystems represent one of the most significant and delicate ecological systems within the global terrestrial landscape, with southwest China housing the world’s largest continuous exposed karst region, encompassing the provinces of Yunnan, Guizhou, and Guangxi (Wang et al., 2019). Since the 1970s, large-scale artificial afforestation has been undertaken in the karst rocky desertification areas of southwestern China, with *Pinus armandii* Franch. (evergreen, recalcitrant litter) and *Alnus nepalensis* (deciduous nitrogen-fixer, labile litter) being the two most prevalent tree species, particularly in the karst faulted basin area situated in the eastern part of Yunnan Province (Tang et al., 2015; Liu et al., 2016; Peng et al., 2023; Zheng et al., 2024; Frietsch et al., 2026). Differences in plant traits, litter quality, and resource strategies between these forests may alter soil microbial community structure and network complexity, leading to distinct SMF mechanisms (Liu et al., 2016; Steinauer et al., 2017; Chen Z. et al., 2025; Hou et al., 2025; Frietsch et al., 2026). Most prior research in karst areas has focused on rocky desertification intensity (Wen et al., 2024), vegetation succession (Xiao C. et al., 2024; Zou et al., 2024), karst types (Liu et al., 2020; Xiao et al., 2022), or individual soil functions. Only a few studies have linked microbial diversity and network complexity to SMF during restoration (Kang et al., 2024, 2025; Long et al., 2024; Xiao C. et al., 2024; Li et al., 2025a). Therefore, examining how soil microbial attributes and network features regulate SMF in different plantation types is crucial for guiding forest management in karst regions and for understanding global forest ecosystem stability.

This study investigates the multidimensional characteristics of soil microbial communities–including diversity, network complexity, and sensitive taxa–and their relationships with SMF in two representative karst plantations (*P. armandii* and *A. nepalensis*) on Liangwang Mountain, central Yunnan. We address three questions: (1) How do soil microbial diversity, network complexity, and SMF differ between the two forests? (2) Which microbial attributes best explain variations in SMF? (3) Which environmental factors shape these microbial attributes and thereby influence SMF? The findings are expected to provide insights for managing and enhancing ecosystem functions in subtropical karst regions.

## 2 Materials and methods

### 2.1 Study site and sampling

This study was conducted at Liangwang Mountain, located in the southeastern part of Chenggong District, Kunmin City (24°72′–24°77′N, 102°86′–102°96′E), China (Supplementary Figure 1), and did not require ethical approval. Liangwang Mountain is situated within the northern subtropical low-latitude plateau monsoon climate zone, characterized by a mild climate, abundant sunlight, and distinct wet and dry seasons. The region experiences synchronous rainfall and heat during the same season, alongside asynchronous light and temperature variations. The elevation ranges from 1800 to 2400 m above sea level. The average annual temperature is 11.7 °C, with annual precipitation ranging from 1100 to 1400 mm, and a relative humidity of 76%. The predominant soil type in this area is red calcareous soil (Calcaric Cambisols, FAO). The region under consideration is an example of a karst ecosystem, distinguished by the presence of limestone bedrock and muddy siltstone formations. Before the 1970s, widespread deforestation and land clearing activities precipitated significant soil and nutrient depletion, culminating in land degradation and severe desertification within the region. In addressing these environmental challenges, the region implemented comprehensive aerial ecological restoration strategies, leading to the establishment of pure plantations characterized predominantly by *Pinus armandii* and *Alnus nepalensis* species. Therefore, the study area encompasses two distinct categories of plantation, namely *Pinus armandii* plantations (PAF) and *Alnus nepalensis* plantations (ANP), as determined through comprehensive field evaluation.

Since the 1970s, aircraft have been employed to disperse seeds of *Pinus armandii* and *Alnus nepalensis* species on barren or abandoned lands affected by rocky desertification. These species are well-adapted to the local environment. Subsequently, these areas have been enclosed, maintained, and managed until the vegetation has been fully restored. Thus, the *Pinus armandii* plantations (PAF) and *Alnus nepalensis* plantations (ANP) have been undergoing regeneration for approximately 60 years following the destruction of the primary forests in 1950. The plants distributed under the PAFs are mainly classified as *Hedychium*, *Oplismenus compositus*, *Ageratina adenophora*, *Polygonum chinense* var. Paradoxum, *Dumasia*, *Pyracantha fortuneana*, *Senecio scandens*, *Myrsine africana*, *Polygonum japonicum*, *Pteridium revolutum*, *Impatiens uliginosa*, *Elaeagnus*, and *Zanthoxylum armatum*. The plants distributed under the ANPs are mainly classified as *Ageratina adenophora*, *Senecio scandens*, *Carex baccans*, *Rubus*, Urticaceae, *Pteridium revolutum*, *Artemisia princeps*, *Coriaria nepalensis* and *Elshotzia rugulosa*.

In November 2021, we established 16 experimental plots, comprising two vegetation types with eight replicates each, each measuring 30 m × 30 m (Supplementary Figure 1). A minimum spatial separation of 1000 m was maintained between individual plots. At each plot, ten soil cores with a diameter of 10 cm were randomly extracted following an “S” pattern after the removal of surface stubble and litter layers. These soil samples, collected to a depth of 30 cm, were subsequently combined to create a composite sample for each plot. In total, 16 composite soil samples were collected. The soil samples were promptly transported to the laboratory in sterile plastic bags. Subsequently, plant fragments were eliminated by sieving the samples through a 2.00 mm mesh. The soil samples were then divided into three portions, each designated for distinct analytical purposes. A portion of the fresh soil sample was preserved at −80 °C for subsequent DNA extraction. Another portion was maintained at 4 °C to facilitate the analysis of soil microbial biomass and associated activities. The remaining portion of each soil sample was permitted to air dry naturally and was subsequently utilized for the analysis of its physical and chemical properties.

### 2.2 Soil physicochemical properties and multifunctionality analysis

The physicochemical and microbial properties of the soil were assessed in accordance with established protocols (Kang et al., 2022), with specific indices detailed in Supplementary Figures 2, 3 and Supplementary Tables 1, 2.

The present study examined five critical soil functions –namely nutrient provisioning, nutrient cycling, soil water retention (WHC), microbial growth efficiency, and soil organic matter decomposition – in order to evaluate soil multifunctionality (SMF). Soil water holding capacity (WHC) – representing the maximum amount of water a soil can retain after free drainage (water holding capacity, WHC) – was selected as a direct measure of the water retention function. WHC was determined using the classic saturated cutting-ring method (followed by oven-drying) as described by Kang et al. (2022). For the assessment of nutrient provisioning, we selected indicators such as soil ammonium nitrogen content (NH4-N), nitrate nitrogen content (NO3-N), available phosphorus (AP), available potassium (AK), exchangeable calcium (ECa), and magnesium (EMg), along with microelements including available iron (Fe), manganese (Mn), copper (Cu), and zinc (Zn) (Supplementary Table 2). These indicators were chosen because they directly reflect the availability of nutrients for plant uptake and microbial activity, which are essential for ecosystem functioning (Hu et al., 2021; Dai et al., 2023). The availability of these nutrients represents the immediate fluxes of nutrients within the soil, rendering them particularly suitable as process-based indicators of nutrient provisioning. Although total soil nutrients (SOC, TN, TP) are not directly indicative of the dynamic processes of soil nutrient supply, they represent the long-term nutrient storage capacity of the soil (Supplementary Table 2). Hence, these parameters are critical attributes that significantly impact ecosystem function (Garland et al., 2021; Li et al., 2025a). The size and composition of these long-term nutrient pools (e.g., SOC, TN) regulate microbial biomass accumulation and respiratory activity, which are key components of microbial growth efficiency. The efficiency of microbial growth was assessed through the quantification of soil microbial biomass carbon (MBC), microbial biomass nitrogen (MBN) and microbial biomass phosphorus (MBP), and soil respiration (SR) (Wang et al., 2023).

Despite the limitations of using βG and βX activities (Supplementary Table 2) as indicators of SOM decomposition, these enzyme activities provide valuable insights into the ability of SOM to supply labile carbon sources for microorganisms (Bai et al., 2024). Therefore, βG and βX activities serve as useful indicators of SOM decomposition (Wang et al., 2023). Dissolved organic carbon (DOC) and dissolved organic nitrogen (DON), and key extracellular enzymes including L-leucine aminopeptidase (LAP), peroxidase (PeO), phenol oxidase (PhO), β-N-acetylglucosaminidase (NAG), acid phosphomonoesterase (ACP), and β-D-Cellobiohydrolase (CBH) activities (Supplementary Table 2) were used to assess nutrient cycling (Li Y. et al., 2023; Kang et al., 2025). The carbon, nitrogen and phosphorus cycling index are detailed in the Supplementary Appendix 1. Ultimately, the averaging method (Garland et al., 2021; Duan et al., 2023; Kang et al., 2024; Long et al., 2024) was utilized to derive the SMF from the 28 selected functional variables, which serves as an important instrument for comprehending complex and interrelated processes (Wagg et al., 2019; Delgado-Baquerizo et al., 2020).

To complement the averaging method and to assess trade-offs or synergies among individual functions, we applied a multithreshold approach (Zhang et al., 2024). The 28 individual functional variables (Supplementary Table 1) were normalized to a 0–1 scale using min-max normalization. A series of thresholds from 0 to 0.95 at intervals of 0.05 was applied. For each sample and each threshold, we calculated the proportion of functions exceeding that threshold. The area under the threshold-multifunctionality curve (AUC) was used as an integrated measure. To evaluate the relative contribution of each function to SMF, we performed a leave-one-function-out analysis: we recalculated the threshold curves after sequentially removing each single function and quantified the reduction in AUC relative to the full set of functions. A larger reduction indicates a stronger contribution. Pairwise Pearson correlations among the 28 functions were also calculated separately for PAF and ANP to examine trade-offs and synergies. All analyses were performed using R (v4.6.0) with custom scripts.

### 2.3 Soil microbial properties analysis

The detailed methodologies for extracting soil genomic DNA are presented in Supplementary Appendix 2. The co-occurrence networks of hierarchical communities were analyzed within the context of ecological restoration using the “WGCNA” package (Langfelder and Horvath, 2008), with extensive information available in Supplementary Appendix 3. The Harman method was employed to determine the sensitive amplicon sequence variation (ASV) between two distinct types of plantations in karst regions (Hartman et al., 2018), with further details provided in Supplementary Appendix 4. In total, 2505, 4310, and 3033 ASVs were identified for bacteria, fungi, and protists, respectively.

### 2.4 Statistical analyses

Analyses were performed using SPSS 25.0 and R (v4.0.2). To test for significant differences (*P* < 0.05) in pH, multiple ecosystem functions, and microbial diversity indices, one-way analysis of variance (ANOVA) with the least significant difference (LSD) test was applied. Data were confirmed to be normally distributed with homogeneous variances, undergoing log transformation as needed. To compare the overall structure of functional correlations between PAF and ANP, we performed a Mantel test (vegan package, R) based on Spearman’s rank correlation matrices of the 6 individual functions (Supplementary Figure 3C). The significance was assessed using 999 permutations. Visualization of shifts in microbial community composition at distinct artificial forests in karst areas was achieved through non-metric multidimensional scaling (NMDS) ordination of Bray-Curtis dissimilarities.

Following the protocols outlined in Li et al. (2022), the mean α- and β-diversity indices were calculated. Following the protocols outlined in Yuan et al. (2021), the vulnerability index was calculated. Linear regression analysis was employed to identify correlations between SMF and microbial variables, including microbial diversity index, Module 3, network complexity and stability, and sensitive taxa. Pearson correlation coefficient analyses were performed to evaluate the degree of association between individual functions, sensitive taxa, and SMF. Heat maps were utilized to visually depict variations in sensitive taxa within Module 3 under the different artificial forests. Random Forest (RF) analysis, conducted using the “randomForest” package in R, was used to identify the key factors influencing the sASVs within Modules, and SMF.

## 3 Results

### 3.1 Effects of the different plantations on soil pH, single functions, and multifunctionality in karst regions

**FIGURE 1:**
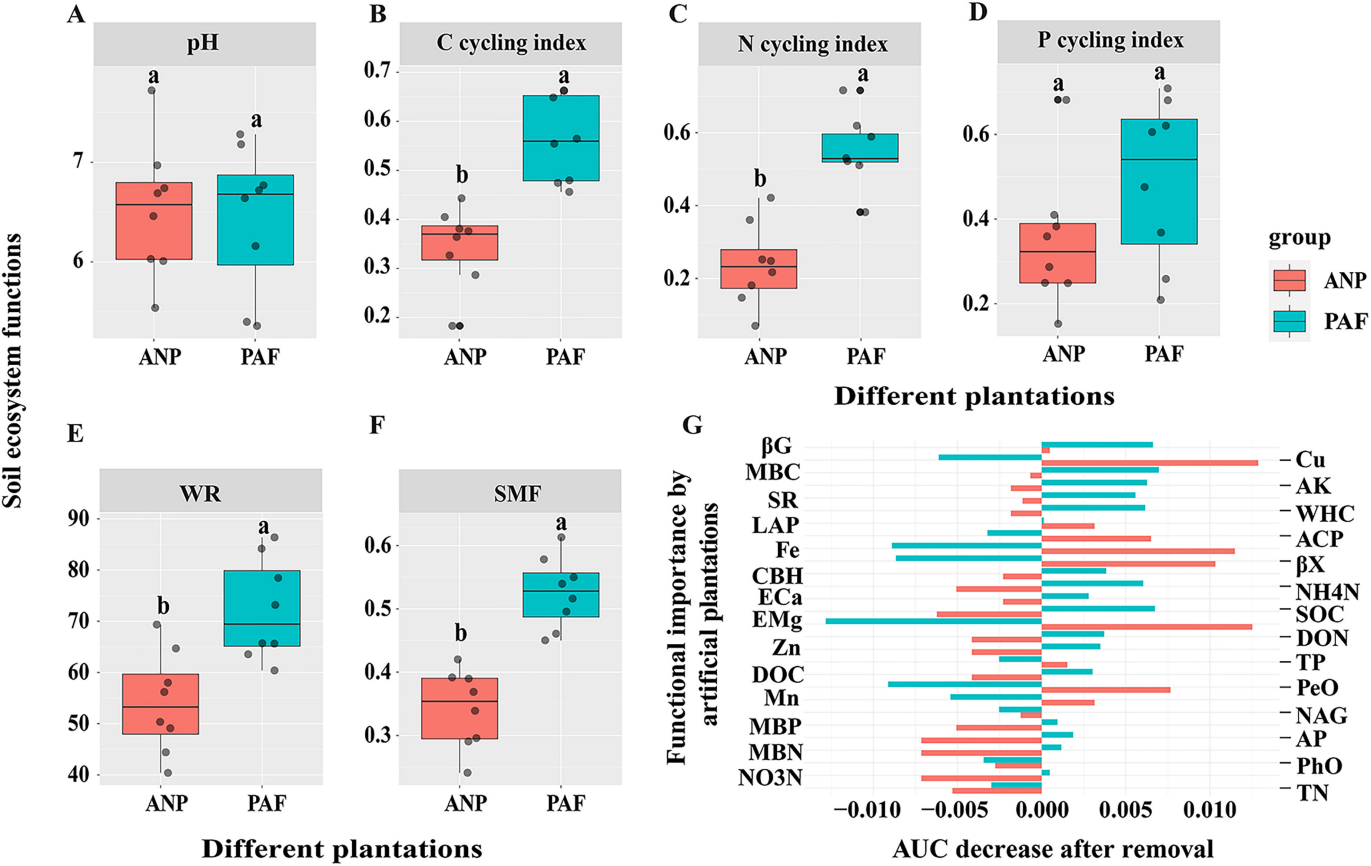
Effects of different plantations restoration on soil functions **(A–E)**, SMF **(F)**, and **(G)** leave-one-function-out importance for PAF (orange) and ANP (blue) in a subtropical karst region. Different letters denote statistically significant disparities among treatments, as determined by the LSD test (*p* < 0.05). Each dot represents individual data (*n* = 8). Positive values indicate that removal reduces multifunctionality; negative values indicate that removal increases multifunctionality (functional redundancy or bottleneck release). ANP, *Alnus nepalensis* plantations; PAF, *Pinus armandii* Franch plantations; WR, water regulation; SMF, soil multifunctionality.

There was no significant difference in soil pH between the two typical plantations (ANP vs. PAF) in the subtropical karst region of southwest China (Figure 1A). Soil carbon cycling, nitrogen cycling, water holding capacity (WHC) and SMF all exhibited significantly higher values in PAF than ANP. Conversely, there was no significant change in phosphorus cycling (*p* < 0.05, Figures 1B–F). Pairwise correlation analyses among those functions showed predominantly positive correlations, and no significant negative trade-offs were observed in either plantation type (Supplementary Figures 3A–C). The correlation patterns were largely stable between PAF and ANP (Mantel test, *r* = 0.68, *p* < 0.01), suggesting that the overall structure of functio-function relationships differs between the two plantations.

### 3.2 Multi-threshold analysis and functional contributions

The multi-threshold approach revealed that PAF consistently exhibited a higher proportion of functions exceeding a given threshold than ANP across the entire threshold range (0–0.95), with the most pronounced differences observed at intermediate thresholds (0.4–0.8; Supplementary Figure 3D). The area under the curve (AUC) was significantly larger in PAF than in ANP (*p* < 0.05, *t*-test), confirming the higher overall multifunctionality of PAF. The leave-one-function-out analysis showed that removal of βG, Cu, MBC, AK, SR, WHC, LAP, ACP, Fe, and βX led to the largest decline in AUC (positive mean_decrease; Supplementary Figure 3E). Among these, βG and MBC were the strongest positive contributors. In contrast, removal of most nitrogen-related functions (NH4-N, NO3-N, TN, DON, MBN) resulted in a slight increase in AUC (negative mean_decrease; Supplementary Figure 3E), indicating functional redundancy or a bottleneck effect, particularly in the ANP group. When the analysis was performed separately for each forest type (Figure 1G), striking differences emerged. In PAF, the positive contributors were primarily carbon-, nitrogen-, water-, and potassium-related functions (SOC, MBC, βG, NH4-N, AK, WHC, SR). In ANP, the positive contributors were micronutrients (Cu, EMg, Fe, Mn) and lignin-/phosphorus-related enzymes (βX, PeO, ACP). Removal of carbon-nitrogen functions in ANP actually increased AUC (negative mean_decrease; Figure 1G), reflecting that these functions are universally low in ANP and act as bottlenecks.

### 3.3 Effects of the different plantations on soil microbial biodiversity and its relationship with SMF

**FIGURE 2:**
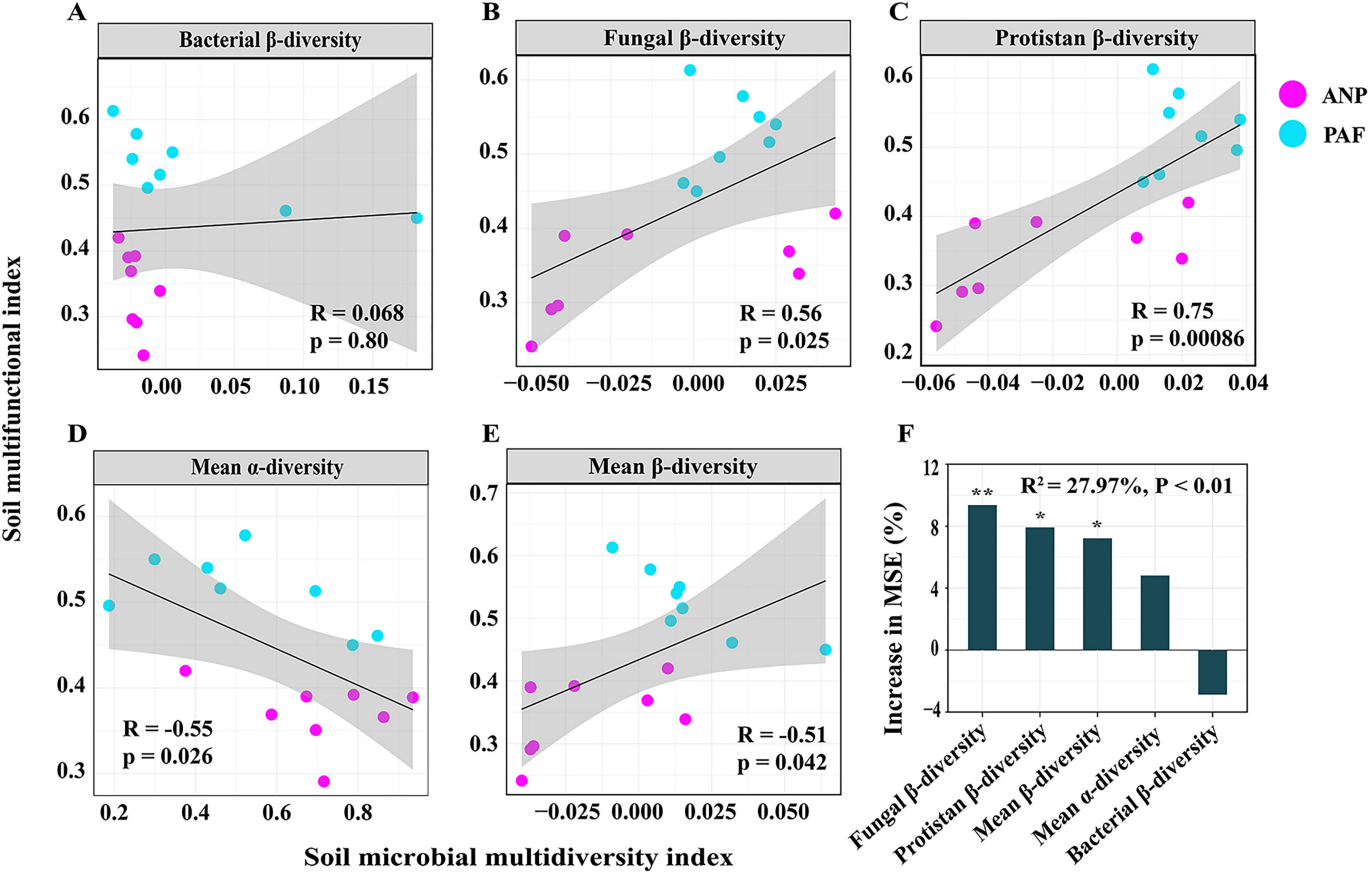
Relationships between soil microbial diversity and SMF following the types of different plantations in a subtropical karst region. **(A–E)** Soil bacterial, fungal, protistan β-diversity, mean microbial α- and β-multidiversity index, respectively. **(F)** Importance of predictors of soil microbial diversity for SMF, based on the random forest mean. ANP, *Alnus nepalensis* plantations; PAF, *Pinus armandii* Franch plantations. *P < 0.05; **P < 0.01.

There were no significant changes in alpha diversity of bacterial, fungal and protist taxa between two typical plantations in the subtropical karst region of southwestern China (Supplementary Figure 4). However, fluctuations in the richness, Chao1, and ACE indexes of fungi and protists exerted a substantial influence on the SMF (Supplementary Figure 5). NMDS revealed significant alterations in fungal and protistan communities between ANP and PAF (PERMANOVA test, *p* < 0.01), while no significant differences were observed in bacterial communities (Supplementary Figure 6). Interestingly, the β-diversity indices for both fungi and protists exhibited a significant positive correlation with SMF, while no such correlation was observed for bacteria (Figures 2A–C). Moreover, the microbial multitrophic α-diversity index exhibited a significant negative correlation with SMF (*p* = 0.026), whereas the multitrophic microbial β-diversity index demonstrated a contrasting relationship (*p* = 0.042, Figures 2D,E). However, the Random Forest (RF) model showed that the SMF was primarily drived by the β-diversity of the fungal community, followed by the β-diversity of protistan communities and the mean microbial β-multidiversity index. In contrast, the multitrophic microbial α-diversity and bacterial β-diversity had lesser impacts (Figure 2F).

### 3.4 Effects of the different plantations on the complexity and stability of soil microbial co-occurrence networks and their association with SMF

**FIGURE 3:**
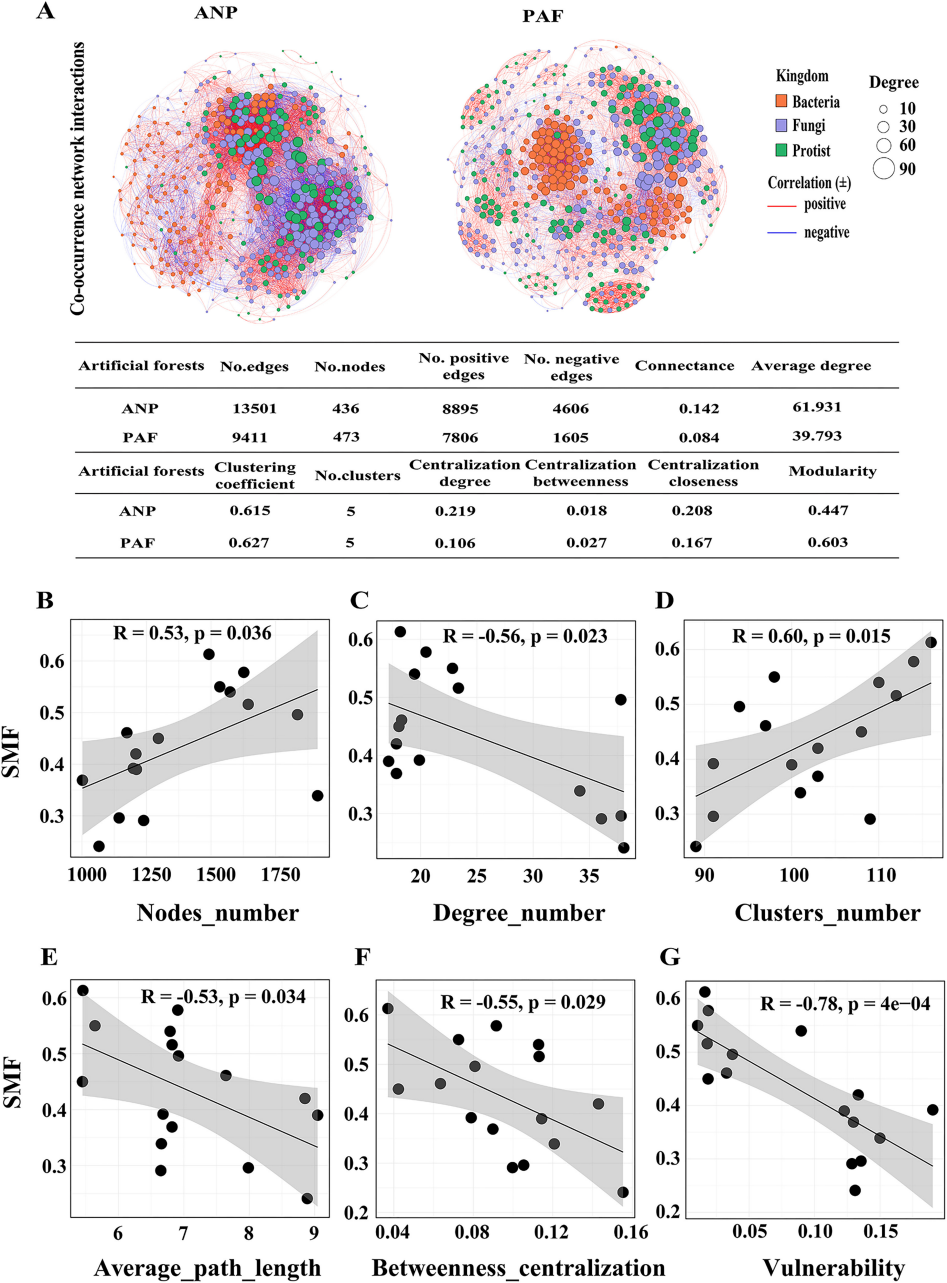
Effects of different plantations restoration on the complexity and stability of co-occurrence networks of soil bacterial, fungal, and protistan communities **(A)** and their relationships with SMF **(B–G)** in a subtropical karst region. The nodes, distinguished by varying colors, correspond to bacterial, fungal, and protistan ASVs, respectively. Each edge signifies a robust and statistically significant correlation between two nodes. Specifically, correlations were deemed robust if they exhibited Spearman correlation coefficients greater than 0.8 and *p*-values less than 0.01, and these were utilized in the network construction, where each node represents an individual ASV. A red line denotes a positive correlation, whereas a green line indicates a negative correlation. The size of each node is proportional to its degree. ANP, *Alnus nepalensis* plantations; PAF, *Pinus armandii* Franch plantations.

The patterns of soil bacterial, fungal and protistan taxa co-occurrence networks exhibit distinct variations under the different plantations in a subrtopical karst region, southwest China (Figure 3A). Notably, in the *Alnus nepalensis* plantations (ANP), the number of edges (both positive and negative), connectance, average degree, and centralization closeness were all significantly higher than that in the *Pinus armandii* Franch plantations (PAF). In contrast, the number of nodes, the clustering coefficienct, the centralization betweenness, and the modularity showed the opposite trend (Figure 3A). This result suggests that the ANP harbored more complex microbial networks than PAF. Moreover, an analysis of the sub-networks generated from the meta-network for each soil sample–by retaining nodes associated with specific samples and all corresponding edges–revealed significant positive correlations between the SMF and both the number of nodes and clusters. In contrast, the degree, average path length, and betweenness centralization exhibited negative correlations (Figures 3B–F). Additionally, a noteworthy negative correlation was established between SMF and the vulnerability (Figure 3G).

### 3.5 Effects of the different plantations on the microbial network patterns and the sensitive ASV

**FIGURE 4:**
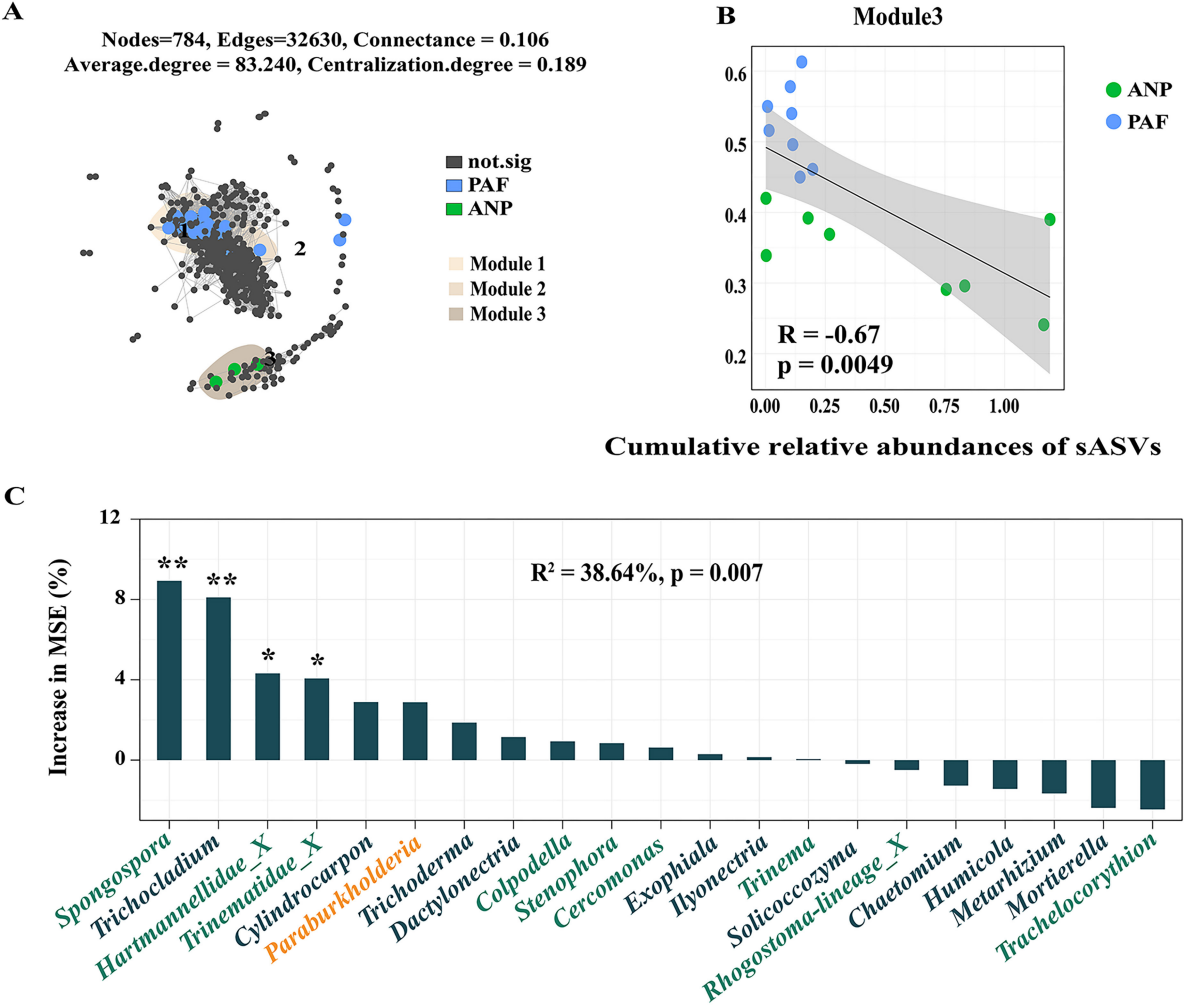
Identification of soil sensitive microbial communities and their contribution to SMF under different plantations in a subtropical karst region. **(A)** Co-occurrence interactions of sensitive amplicon sequence variants (sASVs) in soil microbial communities across various plantations were analyzed. Networks were constructed using robust correlations (Spearman’s > 0.6, *p* < 0.01), with nodes representing ASVs and edges indicating strong, significant correlations. ASVs were color-coded based on their association with karst plantations, while gray ASVs were insensitive to vegetation types. **(B)** The relationship between Modules 3 and SMF in different karst plantations are shown. Black lines indicate ordinary least squares regressions, with shaded areas representing the 95% confidence interval. **(C)** Forest analysis is conducted to assess the significance of the dominant taxa in module 3 in driving SMF. Variable importance was assessed by the percentage increase in mean square error (MSE). ANP, *Alnus nepalensis* plantations; PAF, *Pinus armandii* Franch plantations. **P < 0.01; *P < 0.05.

The identification of three distinct modules within the network suggests that the soil microbial co-occurrence network, along with its ecological modules, has been significantly altered due to the restoration of different plantations in a subtropical karst region (Figure 4A). In Module 1, with a cumulative relative abundance of 99.6%, and Module 2, with a cumulative relative abundance of 100%, the bacterial communities exhibited dominance. Conversely, in Module 3, the sensitive fungal and protistan communities demonstrated dominance, with cumulative relative abundances of 65.5% and 32.7%, respectively (Supplementary Figure 7A). Compared to ANP, PAF showed a lower cumulative relative abundance of sASVs in Module 3 (Supplementary Figures 7B,C). However, only the abundance of sASVs in Module 3 has a negative linear relationship with the SMF (*R* = −0.67, *p* = 0.0049; Figure 4B, Supplementary Figure 7D). In addition, at the genus level, the relative abundance of *Spongospora* (protists), *Trichocladium* (fungi), *Hartmannellidae_X* (protists), and *Trinematidae_X* (protists) were more influential in SMF than other dominant taxa in module 3 based on the RF (R^2^ = 38.64%, *p* = 0.007) and linear regression analyzes (Figure 4C, Supplementary Figures 8, 9).

### 3.6 Key regulators of SMF

**FIGURE 5:**
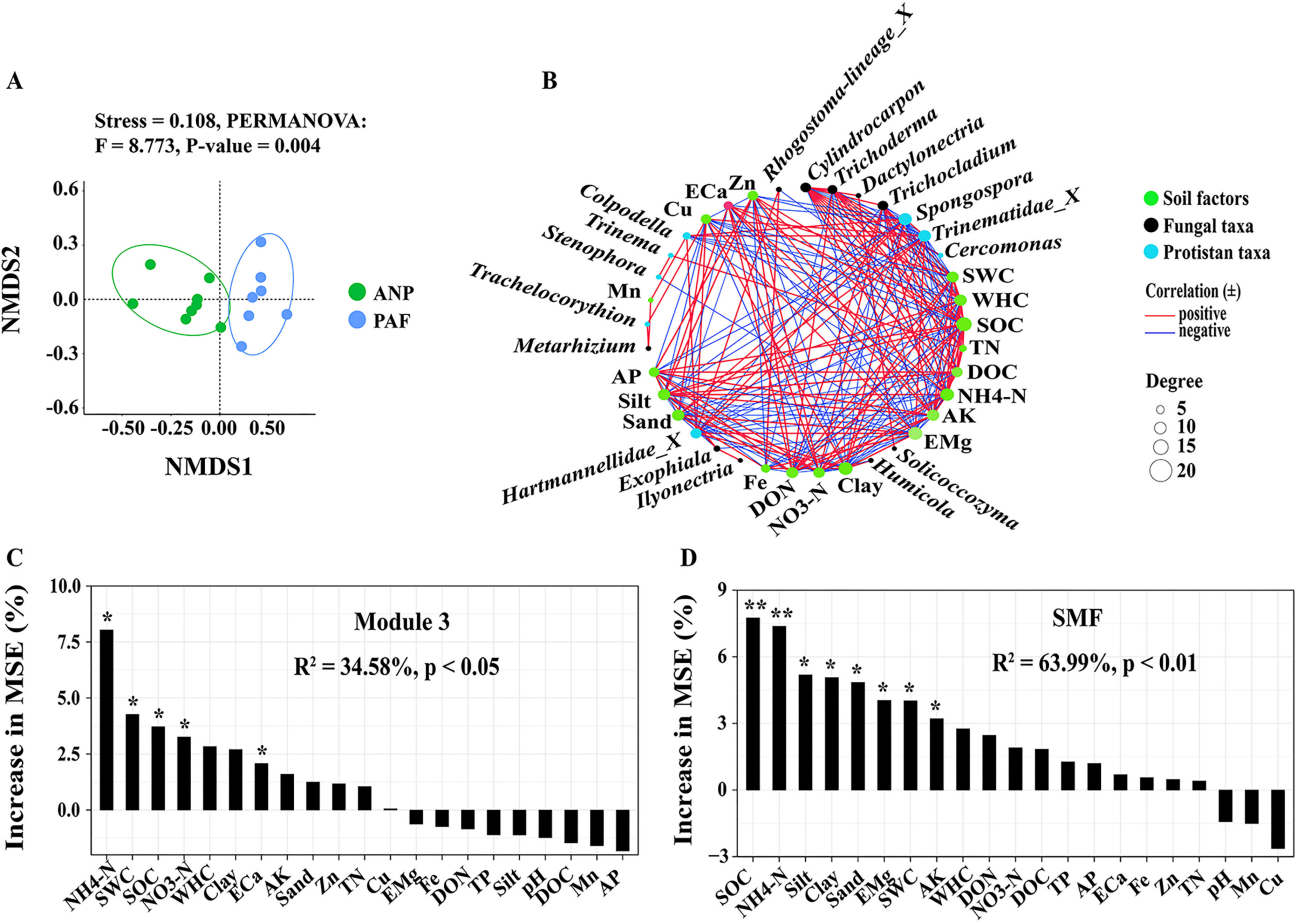
Relationship between soil properties and SMF. **(A)** The modification in the composition of the dominant taxa within the bacterial, fungal, and protistan communities in module 3 was illustrated utilizing non-metric multidimensional scaling (NMDS) plots. **(B)** Network analysis shows the relationships between the dominant taxa of bacterial, fungal and protistan communities in module 3 and soil properties under the different plantations in a subtropical karst region. Node size corresponds to the number of connections (degree), while red and blue lines indicate strong positive and negative linear relationships, respectively. **(C)** Random forest mean predictor importance of the attributes of the soil properties for Module 3, and **(D)** SMF in the different plantations in a subtropical karst region. The significant levels are: *p < 0.05, **p < 0.01. Abbreviations of traits as in Supplementary Figure 2 and Supplementary Table 2.

Non-metric multidimensional scaling analysis indicated that the distribution patterns of dominant sensitive ASVs within module 3 were significantly influenced by the presence of various plantations in a subtropical karst region, as demonstrated by the PERMANOVA test (*F* = 8.773, *p* = 0.004; Figure 5A). Furthermore, a co-occurrence network analysis identified 213 notable interactions between soil physicochemical properties and keystone taxa, with positive associations accounting for 53.9% of these interactions (Figure 5B). The RF analysis explained approximately 34.58% and 63.99% of the variations in the sensitive ASVs in Module 2 and SMF, respectively, and with the contents of SOC, NH4-N and SWC as the common main factors. Additionally, soil texture, AK and EMg were also key abiotic factors in regulating the SMF (Figures 5C,D). Consequently, the influence of these soil factors on sensitive microbial taxa during the restoration of different plantations in a subtropical karst region is pivotal in modulating soil multifunctionality.

## 4 Discussion

### 4.1 Microbial diversity and community composition drive SMF differences

Afforestation is a key restoration strategy in karst regions, enhancing soil quality and ecological functions (Cao and Xiong, 2024; Kang et al., 2025; Lei et al., 2025). While soil microbial diversity often reflects restoration success (Guo et al., 2021), we found no significant differences in bacterial, fungal, or protistan α-diversity between the two plantations (Supplementary Figure 4), consistent with studies in similarly restored karst areas (Lu et al., 2022; Xiao C. et al., 2024). Relationships between microbial diversity and ecosystem functions remain inconsistent across studies (Delgado-Baquerizo et al., 2020; Gong et al., 2024; Hu et al., 2024; Wang et al., 2024; Kang et al., 2025; Lei et al., 2025; Li et al., 2025b; Lu et al., 2025; Luo et al., 2025; Sun et al., 2025). Here, fungal and protistan α-diversity (Richness, Chao1, ACE) correlated negatively with SMF, whereas bacterial α-diversity showed no correlation (Supplementary Figure 5), possibly due to group-specific functional roles or differences in functional redundancy and niche complementarity (Han et al., 2021; Yang et al., 2023).

Besides α-diversity, microbial community composition is crucial in regulating soil functions (Chen et al., 2023; Hu et al., 2024; Kang et al., 2024). NMDS revealed significant differences in fungal and protistan communities between plantations, but not in bacterial composition (Supplementary Figure 6). Intriguingly, SMF correlated positively with fungal and protistan β-diversity (Figure 2), indicating that shifts in community structure–particularly of fungi and protists–drive SMF variation (Figure 2). Multi-trophic microbial β-diversity also positively contributed to SMF (Figure 2), aligning with previous work (Li et al., 2022; Wang et al., 2024), whereas multi-trophic α-diversity had minimal influence. Together, these findings suggest that differences in SMF between plantations are primarily mediated by changes in microbial community structure, especially of fungi and protists, rather than by bacterial communities or α-diversity alone.

The multi-threshold analysis further substantiated the higher SMF in PAF and revealed that the drivers of SMF differ fundamentally between the two plantations (Figure 1G, Supplementary Figures 3B–E). In the resource-rich PAF, carbon- and nitrogen-based processes (SOC, MBC, βG, NH4-N, AK, WHC) jointly sustain multifunctionality, which aligns with the positive correlations between SMF and fungal/protistan β-diversity and the negative correlation with network complexity (Figures 2, 3). In contrast, in the nutrient-poor ANP, micronutrients and lignin-degrading enzymes become the limiting factors, and the universally low levels of carbon and nitrogen functions act as bottlenecks (Figure 1G, Supplementary Figures 3B–E). The absence of negative trade-offs among functions suggests that plantation management aimed at improving one function is unlikely to impair others. Overall, the complementary multi-threshold approach not only confirms the averaging-based results but also provides mechanistic insights into which functions drive SMF and how they vary with plantation type. These findings are consistent with the random forest results presented that SOC, NH4-N, and SWC were identified as key abiotic drivers (Figure 5).

### 4.2 Microbial network complexity, modules, and their links to SMF

Soil micro-food webs, comprising bacteria, fungi, and protists, play integral roles in sustaining SMF (Bardgett and van der Putten, 2014; Delgado-Baquerizo et al., 2020; Zhu et al., 2024). While complex microbial networks are often associated with higher SMF in various ecosystems (Wagg et al., 2019; Chen et al., 2022; Li S. et al., 2023; Wang et al., 2024; Luo et al., 2025), we found a negative correlation between network complexity (e.g., degree, betweenness centrality) and SMF (Figure 3). This aligns with studies noting that reduced network complexity can support higher SMF during restoration (Xiao et al., 2022; Chen et al., 2023). A potential explanation is that the soil organic carbon content and nutrient availability in *Alnus nepalensis* plantations (ANP) are comparatively lower than those in *Pinus armandii* Franch plantations (PAF) (Supplementary Table 2), suggesting that the ANP ecosystems experience increased stress due to nutrient-poor conditions. As a result, the microbial communities within the ANP may necessitate greater complexity to sustain their stability (Lin et al., 2021; Chen et al., 2023).

Another an alternative explanation can be derived from the observation by Lin et al. (2021), which posits that taxa with positive correlations often exhibit analogous responses to environmental changes, leading to “co-oscillation” within the community and consequently destabilizing networks. Our research demonstrates that the proportion of positive correlations in microbial co-occurrence networks is higher in the PAF compared to the ANP (Figure 3). This implies that the former experiences more pronounced “co-oscillation” within its community, thereby resulting in diminished network stability. Thus, our findings further confirmed that there is a strong negative correlation between SMF and the vulnerability of the bacterial-fungal-protistan co-occurrence network (Figure 3). Additionally, given the intricate nature of karst ecosystems, the positive correlation between the complexity of microbial hierarchical networks and SMF is not consistently robust (Li et al., 2022; Chen et al., 2023; Li Y. et al., 2023; Kang et al., 2024, 2025). Accordingly, it is essential to acknowledge that the complexity of microbial hierarchical networks is pivotal in the restoration of ecological functions.

Modularity in co-occurrence networks helps reveal ecological linkages not captured by diversity metrics alone (Jiao et al., 2020). Modules often group taxa with similar ecological roles (Delgado-Baquerizo et al., 2020) and can predict SMF (Li et al., 2022; Yang et al., 2023; Xiao Y. et al., 2024; Zhang et al., 2024). However, in our present work, a significant negative correlations between the abundances of taxa in module 3 and SMF was observed (Figure 4, Supplementary Figure 7). These results were supported by Li et al. (2022) and Yang et al. (2023). A plausible explanation is that this module may predominantly utilize soil organic matter in the ANP compared to the PAF, thereby contributing to a reduction in soil organic carbon (Supplementary Table 2), which may further lead to strong negative correlations between Module 3 and soil multifunctionality. Additionally, taxa across different modules (e.g., Modules 1 and 2) exhibit reduced interactions, likely attributable to habitat isolation, which consequently impedes the coexistence of microbial species (Li et al., 2022). In contrast, Modules 1 and 2 (bacteria-dominated) showed no significant correlation with SMF (Figure 4, Supplementary Figure 7), likely due to functional redundancy within bacterial communities (Xiao Y. et al., 2024). Overall, microbial co-occurrence patterns modulate the diversity–multifunctionality relationship in these karst plantations.

From a mechanistic perspective, the negative correlation between microbial network complexity and soil multifunctionality (SMF) can be interpreted using niche theory and the stress-gradient hypothesis (Adams et al., 2022; Li et al., 2026). In resource-limited or environmentally stressed systems, such as the karst plantations studied here, microorganisms may intensify competition for limited carbon, nitrogen, and water resources. Under these conditions, increased network complexity–reflected by higher degrees, average path length, and betweenness centrality–does not necessarily indicate functional complementarity (Figure 3); instead, it may reflect intensified antagonistic or competitive interactions, leading to reduced overall ecosystem functioning. According to the stress-gradient hypothesis, positive interactions (e.g., facilitation) are expected to dominate under harsh conditions, but our results show that higher SMF is associated with lower network complexity and vulnerability (Figure 3). This suggests that in the less resource-limited *Pinus armandii* Franch plantations (PAF), microbial communities may achieve high multifunctionality through streamlined, stable networks rather than through redundant or overly interconnected structures. Conversely, in the more nutrient-poor *Alnus nepalensis* plantations (ANP), elevated network complexity may represent an adaptive but energetically costly response to environmental stress, ultimately constraining SMF (Figures 1, 3). Thus, we argue that high network complexity is not universally beneficial; its ecological consequences depend critically on resource availability and environmental context, a perspective often overlooked in biodiversity–ecosystem functioning studies.

### 4.3 Key microbial taxa and abiotic drivers of SMF

**FIGURE 6:**
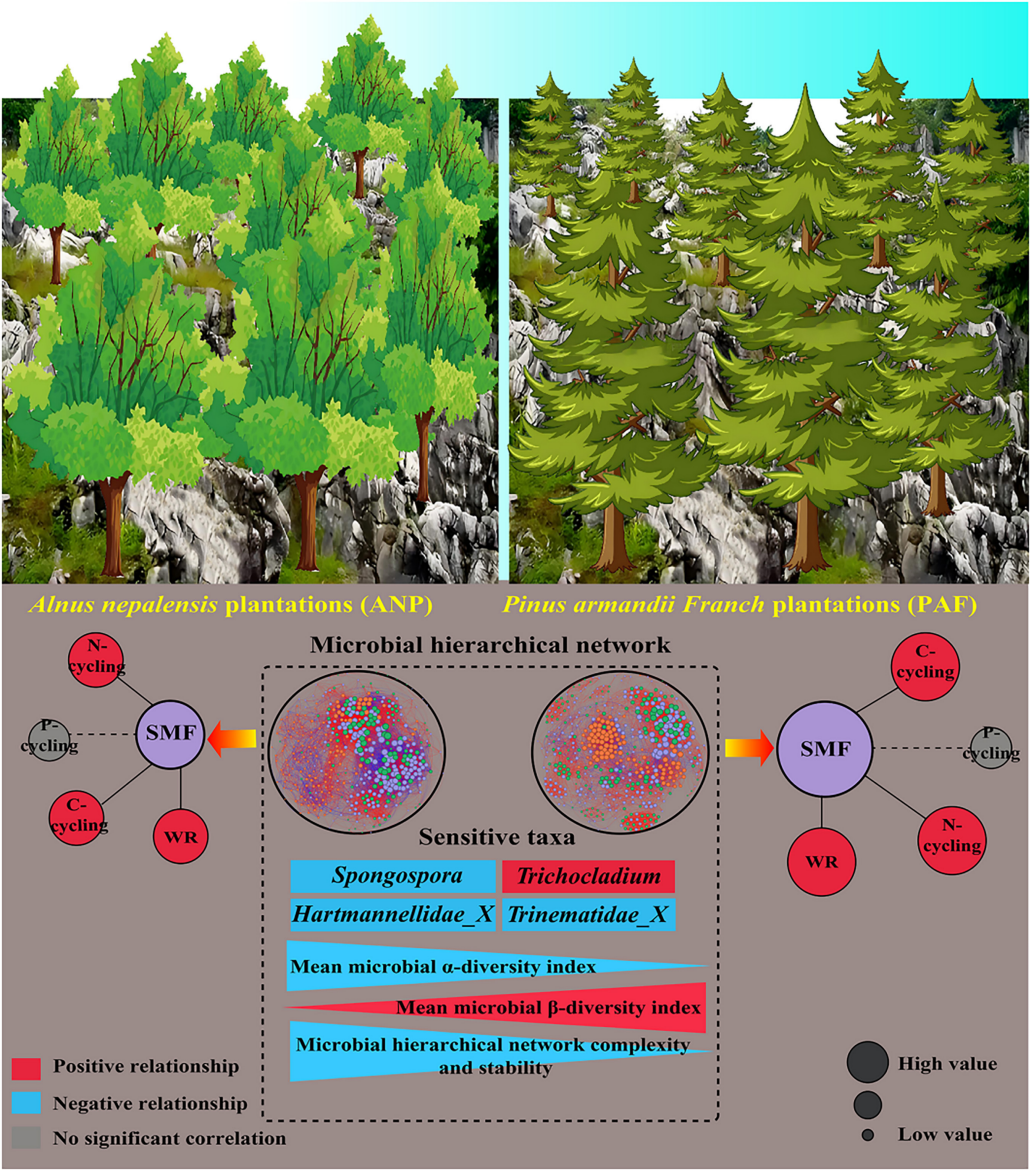
A conceptual framework is presented that illustrates the responses of soil functions, SMF, microbial hierarchical interactions, and keystone taxa to different plantations in a subtropical karst region. The progressively narrowing blue and red triangles, along with their orientation, indicate a reduction in variables. The red and blue rectangles denote significant positive and negative associations with the SMF, respectively. The size of each differently colored circle represents the variation in the value of individual soil functions across different types of plantation in the subtropical karst region. Abbreviations of traits as in Supplementary Figure 2 and Supplementary Table 2.

Specific microbial taxa within ecological modules can be strong predictors of SMF (Zhang et al., 2024). Random Forest regression analysis has been shown to be an effective method for identifying specific microbial taxa that serve as significant predictors of SMF (Kang et al., 2024; Zhang et al., 2024; Li et al., 2025a). According to the findings, one fungal genus (*Trichocladium*) and three protistan genera (e.g., *Spongospora*, *Hartmannellidae_X* and *Trinematidae_X*) were identified within Module 3, contributing 38.64% to SMF (Figures 4, 6). This suggests that the dominant taxa involved in interactions within the ecological clusters of the bacterial-fungal-protistan community associated with different karst plantations were exclusively members of the fungal or protistan communities, rather than the bacterial community.

Specifically, our findings revealed that *Trichocladium* (fungi) was identified as a positive predictor of SMF, while *Spongospora*, *Hartmannellidae_X* and *Trinematidae_X* (protists) were all identified as negative predictors of SMF (Figure 6, Supplementary Figures 8, 9). Despite the limited scope of research conducted thus far on *Trichocladium* (Uke et al., 2021), it is classified within the *Ascomycota* phylum. *Ascomycota* is crucial for breaking down soil persistent organic matter (e.g., cellulose and hemicellulose), and its reduced presence may be linked to declining soil organic matter and fertility (Tedersoo et al., 2014; Zhou et al., 2020). These findings are further corroborated by the current results, which demonstrate that soil organic carbon (SOC) and fertility (Figure 1, Supplementary Figure 2, and Supplementary Table 2), as well as the relative abundance of *Trichocladium* (Supplementary Figure 10), were significantly higher in the PAF compared to the ANP. *Spongospora*, *Hartmannellidae_X* and *Trinematidae_X* are belong to the phylum of *Cercozoa*, which encompasses most known protistan consumers (e.g., bacterivores and omnivores) (Geisen et al., 2018; Sun et al., 2021). Thus, the genera *Spongospora*, *Hartmannellidae_X*, and *Trinematidae_X* potentially play a significant role in modulating bacterial and fungal communities through their grazing activities (Geisen et al., 2018; Sun et al., 2021). Grazing by protists can release nutrients (Chen et al., 2007), but excessive predation may reduce beneficial microbial populations. The negative correlation between these protistan genera and SMF in ANP (Figure 4) therefore suggests that in resource-poor conditions, protist grazing might over-suppress key decomposers, ultimately constraining multifunctionality. In this study, our NMDS analysis indicated that the patterns observed in the fungal and protistan communities were remarkably similar, implying that the structuring of the fungal community is likely influenced by the protistan community (Supplementary Figure 6). This phenomenon was primarily attributed to the role of fungi as the principal prey for protists, which may stimulate fungal metabolic activity and alter the composition of the fungal community. Additionally, In the PAF, the relative abundance of *Spongospora*, *Hartmannellidae_X*, and *Trinematidae_X* was significantly lower than in the ANP (Supplementary Figure 10), suggesting that the lower abundance of carbon-degrading preys (e.g., bacterial and/or fungal taxa) contributes to a higher soil multifunctionality in the PAF (Figure 1). Collectively, these findings substantiate the idea that fungal and protistan communities, as opposed to bacterial communities, are the primary drivers of soil multifunctionality within subtropical karst plantations ecosystems.

Afforestation represents a viable approach for addressing soil erosion, augmenting carbon sequestration, and mitigating the impacts of climate change in regions affected by karst desertification (Wang et al., 2019). The variation in growth characteristics, adaptive strategies, litterfall, and root exudates among different types of plantation results in significant modifications to soil physicochemical properties and the composition of microbial communities (Li S. et al., 2023; Lei et al., 2025). Inconsistent with most previous findings (Chen et al., 2023; Duan et al., 2023; Hu et al., 2024; Luo et al., 2025), the current case study indicated that soil pH was not a significant determinant of the observed variations in the relative abundance of dominant taxa in Module 3 and EMF (Figure 5, Supplementary Figure 3). This suggests that the pH of soil may have the capacity to exert an effect on microbial populations, thereby exerting a concomitant influence on SMF through alterations in substrate efficacy. Soil nutrients, including nitrogen, phosphorus, and potassium, are vital for plant growth in plantations (Che and Jin, 2024). Our findings highlight the importance of SOC, NH4-N, NO3-N, as well as EMg and ECa in influencing the abundance of dominant taxa within Module 3, and EMF (Figure 5), consistent with recent studies (Xiao C. et al., 2024; Kang et al., 2025; Li et al., 2025a). Additionally, recent studies show that moisture directly boosts soil multifunctionality by enhancing activity and interactions among multitropic taxa in micro-food webs, and indirectly by changing microbial community composition (Li S. et al., 2023; Wang et al., 2024; Kang et al., 2025). In line with previous findings, the results of our RF analysis indicated that soil water content also has an important effect on soil multifunctionality in different plantations in a subtropical karst region (Figure 5, Supplementary Figure 3). In conclusion, the biotic and abiotic properties of soil are essential determinants of ecosystem functions within subtropical karst plantations. Concurrently, the pivotal role of functional modules, constituted by soil microorganisms with analogous ecological preferences, in modulating ecosystem functions warrants significant attention.

Overall, our study provides a comprehensive assessment of soil multifunctionality under the different types of plantation in a subtropical karst mountain, offering novel insights into the regulatory roles of multiple microbial parameters (e.g., diversity, composition, modules, dominant sensitive taxa, and network complexity and stability, Figure 6). However, the consideration of several limitations is necessary for the interpretation of our findings. First, this study relies exclusively on a single sampling event, which cannot capture seasonal or interannual variations in soil microbial communities and multifunctionality. Given the strong wet-dry seasonality in subtropical karst regions, the observed relationships among microbial diversity, network complexity, and SMF may vary across different seasons or years. Therefore, future research should adopt time-series sampling strategies – for example, repeated measurements across multiple seasons and years – to unravel the dynamic co-evolution of microbial food webs and multifunctionality during long-term restoration. Second, we did not measure several environmental variables that could potentially influence SMF, such as soil temperature, litter input (quantity and quality), and microbial physiological constraints (e.g., C:N:P stoichiometry of microbes or soil). These factors are known to affect microbial activity and network dynamics (Chen S. et al., 2025; Wang C. et al., 2025). Their absence may limit the explanatory power of our current models, although the random forest analysis already explained 63.99% of the SMF variation using the measured variables (SOC, NH4-N, SWC, EMg, AK). Future studies should integrate these additional regulators to achieve a more comprehensive understanding. Third, the Liangwangshan Ecological Restoration Area in central Yunnan, chosen as the focus of this study, predominantly comprises two types of air-seeded plantations: the *Pinus armandii* Franch plantations (PAF) and *Alnus nepalensis* plantations (ANP). Consequently, mixed plantations dominated by these plantation types are relatively uncommon. As a result, the effects of such mixed plantations on soil ecological multifunctions have yet to be thoroughly investigated. Fourth, we utilized the averaging approach to assess the soil multifunctionality index, which effectively identifies consistent trends across various functions. Nevertheless, this method demonstrates limited efficacy in detecting trade-offs among functions exhibiting opposing trends (Wan et al., 2022). Consequently, future research should incorporate the multiple threshold approach to address this limitation (Zhang et al., 2024; Luo et al., 2025). Notwithstanding this limitation, the present study establishes a theoretical framework for the sustainable management and conservation of karst forests within the region.

## 5 Implications

We explored the relative effects of soil multitrophic microbial α- and β-diversity, and network complexity and stability on soil multifunctionality under the different plantations in a subtropical karst region. The elevated soil carbon cycling index, nitrogen cycling index, and water-holding capacity (WHC) observed in the *Pinus armandii* Franch plantations (PAF) contributed to a significantly greater soil multifunctionality index in comparison to the *Alnus nepalensis* plantations (ANP). This variation is primarily determined by the α-diversity and community structure of fungi and protists, alongside the community structure of multitrophic microorganisms, as well as the complexity and stability of their co-occurrence networks. Furthermore, the pronounced differences in soil multifunctionality observed between the two plantations were attributed to the dominant sensitive taxa within fungi and protists in Module 3, rather than to bacterial communities. Overall, our findings offer a novel perspective on the predictive capacity of potential shifts in belowground communities concerning soil function in planted stand. Additionally, these findings hold significant implications for sustainable plantation or forest management practices.

## Data Availability

The datasets presented in this study can be found in online repositories. The names of the repository/repositories and accession number(s) can be found below: https://ngdc.cncb.ac.cn/gsub/submit/gsa/subCRA055697/finishedOverview, PRJCA 051857, https://ngdc.cncb.ac.cn/gsub/submit/gsa/subCRA055695/finishedOverview, PRJCA051847, https://ngdc.cncb.ac.cn/gsub/submit/gsa/subCRA055678/finishedOverview, PRJCA051835.
