# Supplementary material for: Micro-food web complexity and stability drive differences in soil multifunctionality of subtropical karst plantations in southwest China
Source: Front Microbiol. 2026 Jul 10;17:1845498. doi: 10.3389/fmicb.2026.1845498 (PMC13395863; doi:10.3389/fmicb.2026.1845498)
Supplement: Supplementary file 1 [file Table_1.docx]

**Supplement information**

**Soil micro-food web complexity and stability drive differences in soil multifunctionality of subtropical karst artificial forests in southwest China**

Yalong Kang^1^, Canfeng Li^2^, Linjun Shen^3,^, Yong Huang^4*^

1 International Joint Laboratory for Resource Utilization of Agricultural Solid Waste in Yunnan Province, College of Resources and Environmental Science, Yunnan Agricultural University, Kunming 650201, China

2 Kunming Natural Resources Comprehensive Survey Center of China Geological Survey/Technology Innovation Center for Natural Ecosystem Carbon Sink, Ministry of Natural Resources, Kunming, China

3 School of Ecology and Environmental Sciences; Yunnan University, Kunming, 650500, China

4 School of Ecology and Nature Conservation, Beijing Forestry University, Beijing, China

* Corresponding author: Dr. Yong Huang Email: huangyong@bjfu.edu.cn

**Contents:**

Appendix S1-S4.

Table S1-Table S2.

Figure S1-Figure S10.

**Appendix S1.** **Soil nutrient cycling index calculation**

The study considered multiple nutrient cycling indices, including those for carbon (C), nitrogen (N), phosphorus (P), and other nutrients, as outlined in Table S1. The nitrogen cycling index included MBN, TN, DON, NH4-N, NAG, NO3-N, LAP. The soil's capacity for water regulation was evaluated using the water holding capacity (WHC). The carbon cycling index was assessed through variables such as SOC, DOC, MBC, SR, βG, βX, CBH, PeO, and PhO. The phosphorus cycling index comprised TP, AP, ACP. Other nutrient cycling indices included available potassium (AK), exchangeable magnesium (EMg), exchangeable calcium (ECa), iron (Fe), manganese (Mn), copper (Cu), and zinc (Zn). Each index was calculated by averaging the Z-scores of the respective variables, with scores ranging from 0 to 1 (Maestre et al. 2012, Duan et al. 2023).

**Appendix S2.** **DNA extraction and PCR amplicon sequencing**

Approximately 0.5 g of fresh soil, calculated as dry weight equivalent, was employed for the extraction of total soil genomic DNA using the PowerMax Soil DNA Isolation Kit (MO BIO). The quality of the extracted DNA was evaluated using a NanoDrop 2000 ultra-micro spectrophotometer (Thermo Scientific, USA). The primers and detailed methodology for high-throughput sequencing of bacterial, fungal, and protistan communities followed established protocols (https://doi.org/10.1016/j.jenvman.2024.121395, Appendix A. Supplementary data) (Kang et al. 2024). Data homogenization was performed across all samples, with the sample containing the least amount of data serving as the baseline for homogenization.

**Appendix S3.** **Co-occurrence network construction**

For network construction precision, only ASVs present in at least three subsamples per karst artificial forest and with relative abundance ≥ 0.01% were retained. Associations meeting p > 0.70 and p < 0.05 thresholds were included. The igraph package (Deng et al. 2012) calculated network topology parameters (nodes, edges, degree). Microbial co-occurrence networks for the two artificial forests were then generated and visualized in Gephi.

**Appendix S4.** **Sensitive ASVs and keystone taxa**

The construction of microbial co-occurrence networks and the identification of specialized ASVs (sASVs) and core microbiota adhered to the protocol of Li et al. (2022), implemented using the "igraph" and "Indicspecies" packages. Topological and descriptive metrics, such as numbers of nodes and edges, and degree values, were computed (Li et al. 2022). To define the core microbiota, ASVs were selected based on ubiquity (occurrence in ≥80% of samples) and high relative abundance (ranking within the top 50 most abundant) (Delgado-Baquerizo et al. 2018, Li et al. 2022). Subsequent to filtering out low-frequency ASVs (relative abundance < 0.001%), networks were built and visualized based on significant Spearman rank correlations (r > 0.7, p < 0.05). Identification of keystone taxa employed a dual threshold approach: degree ≥ 150 and closeness centrality > 0.3 (Gao et al. 2021, Xiong et al. 2021, Li et al. 2023). As outlined by Ma et al. (2016), the subgraph function in igraph was then used to extract subnetworks representing each individual soil sample from the overarching mother network of microbiome interactions (Ma et al. 2016).

**Table S1.** **Indicators used to evaluate soil multifunctions in different artificial forests in a subtropical karst region.** SOC, soil organic carbon; DOC, dissolved organic carbon; DON, dissolved organic nitrogen; NH4-N, soil ammonium nitrogen content; NO3-N, nitrate nitrogen content; AP, available phosphorus; TN, total nitrogen; TP, total phosphorus; MBC, microbial biomass carbon; MBN, microbial biomass nitrogen; MBP, microbial biomass phosphorus; SR, soil respiration; βG, β-D-glucosidase activity; βX, β-1,4-xylosidase activity; LAP, L-leucine aminopeptidase activity; PeO, peroxidase activity; PhO, phenol oxidase activity; NAG, β-N-acetylglucosaminidase activity; ACP, acid phosphomonoesterase activity; CBH, β-D-Cellobiohydrolase activity; WHC, soil water retention.

| Individual function | Indicator |
| --- | --- |
| Carbon cycling | SOC, DOC, MBC, SR, βG, βX, CBH, PeO, PhO |
| Nitrogen cycling | TN, DON, NH4-N, NO3-N, MBN, NAG, LAP |
| Phosphorus cycling | TP, AP, ACP |
| Water regulation | WHC |

**Table S2. Changes in soil physicochemical properties in the different plantations in the subtropical karst region.** SOC, soil organic carbon; TN, total nitrogen; NO3-N, nitrate-nitrogen; AP, available phosphorus; DOC, dissolved organic carbon; NH4-N, ammonium nitrogen; SWC, soil water content; TP, total phosphorus; AK, available potassium; EGa, exchangeable sodium concentration; EMg, exchangeable magnesium concentration. The distinct letters in each column represent statistically significant differences between the three treatments determined by LSD at a significance level of α = 0.05. Values are means (n = 8) ± (SE). ANP, *Alnus nepalensis* plantations; PAF, *Pinus armandii* Franch plantations.

| Treatments | Sand | Silt | Clay | SWC | SOC | TN | DOC |
| --- | --- | --- | --- | --- | --- | --- | --- |
|  | (%) | (%) | (%) | (%) | (g kg^-1^) | (g kg^-1^) | (mg kg^-1^) |
| ANP | 53.04±0.73a | 28.08±0.88b | 18.88±0.50a | 54.07±3.50a | 34.21±1.53b | 2.49±0.26a | 212.55±12.20b |
| PAF | 24.21±2.56b | 67.32±2.59a | 8.48±0.52b | 38.43±2.67b | 55.56±2.20a | 3.38±0.37a | 321.84±23.52a |
| Treatments | DON | NH4-N | NO3-N | TP | AP | AK | EGa |
|  | (mg kg^-1^) | (mg kg^-1^) | (mg kg^-1^) | (g kg^-1^) | (mg kg^-1^) | (mg kg^-1^) | (cmol kg^-1^) |
| ANP | 61.63±3.03b | 9.25±0.89b | 8.50±1.64b | 0.77±0.14a | 1.14±0.13b | 269.05±20.87b | 2576.85±144.17b |
| PAF | 85.59±4.28a | 18.35±1.77a | 19.81±3.02a | 0.86±0.14a | 2.67±0.35a | 380.73±18.12a | 3290.37±184.14a |
| Treatments | EMg | Fe | Mn | Cu | Zn |  |  |
|  | (cmol kg^-1^) | (mg kg^-1^) | (mg kg^-1^) | (mg kg^-1^) | (mg kg^-1^) |  |  |
| ANP | 731.10±45.75a | 7.17±0.53a | 80.94±6.56a | 2.73±0.16a | 7.16±0.52b |  |  |
| PAF | 426.02±22.20b | 4.63±0.52b | 77.27±4.48a | 1.97±0.18b | 11.91±1.06a |  |  |

**Figure S1.** Location of the sampling site at Liangwangshan, Kunming City, Yunnan province, China. Two distinct afforestations serve as representative examples of ecological restoration in areas affected by karstic rocky desertification. ANP, *Alnus nepalensis* plantations; PAF, *Pinus armandii Franch* plantations.

**
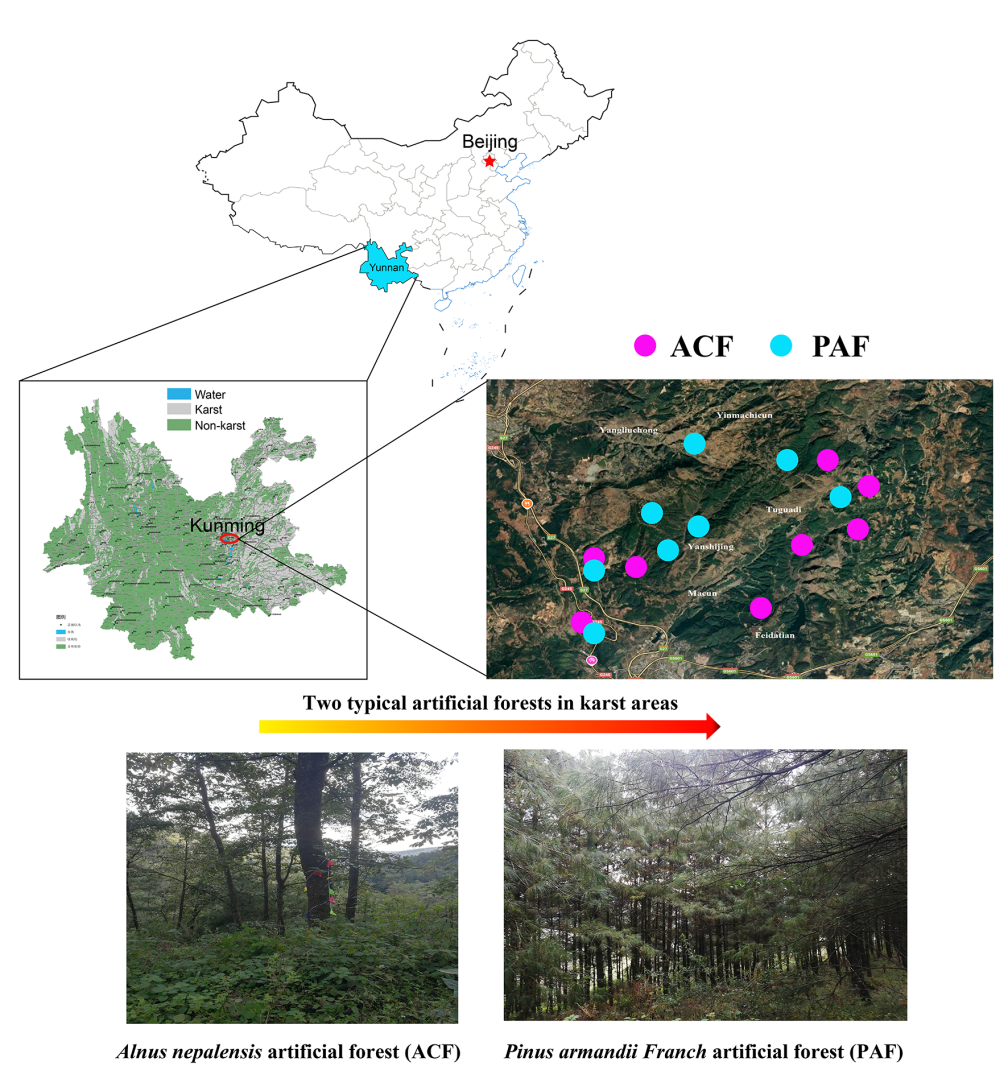
**

**Figure S2.** **Effects of restoration of different karst plantations on soil microbial activities.** Different letters denote statistically significant disparities among treatments, as determined by the LSD test (p < 0.05). The same applies below. ANP, *Alnus nepalensis* plantations; PAF, *Pinus armandii Franch* plantations. BMC, soil microbial carbon; BMN, soil microbial nitrogen; SR, soil respiration; βG, β-1,4-Glucosidase; βX, β-1,4-xylosidase; CBH, β-D-Cellobiohydrolase; LAP, Leucine amino peptidase; NAG, β-1,4-N-Acetyl-glucosaminidase; ACP, Acid phosphomonoesterase; PeO, peroxidase; PhO, Phenol oxidase.

**
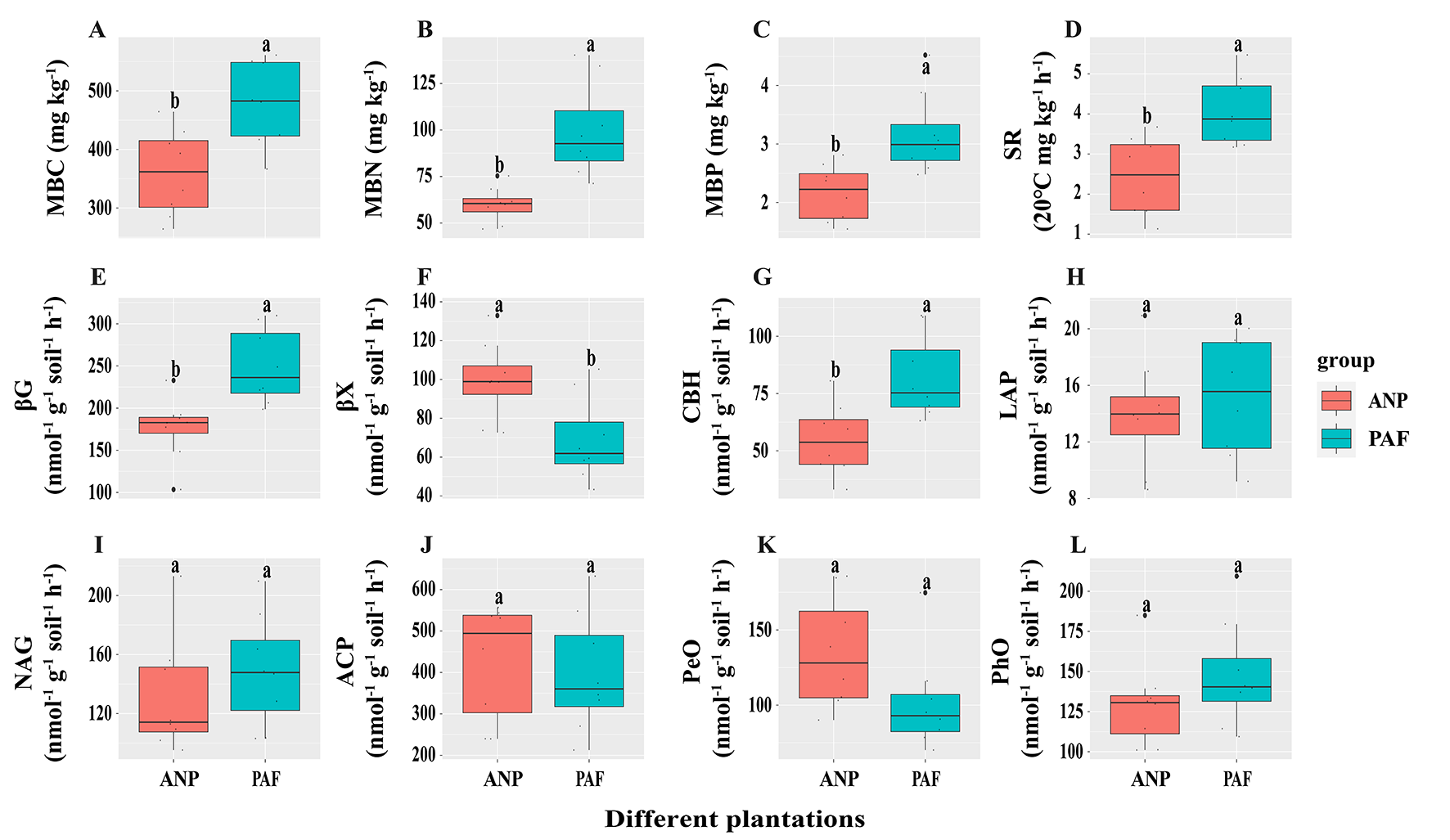
**

**Figure S3. Relationships between soil properties and SMF according to the results from Spearman's correlation analysis (A-B). Pairwise Spearman correlation matrix of the 28 individual functions for PAF and ANP (C).** The blue and red ellipses represent positive and negative relationships among the variables, respectively. The lack of ellipse filling signifies the absence of a significant correlation. * p < 0.05; ** p < 0.01. **Multifunctionality threshold curves for PAF and ANP (D).** Solid lines represent mean proportion of functions exceeding each threshold (n=8 per group); shaded ribbons indicate ± standard error. The higher curve in PAF indicates greater capacity to sustain multiple high-level functions simultaneously. **Importance of individual functions for soil multifunctionality (E).** Bars: mean decrease in AUC after function removal (positive = key driver, negative = redundancy/bottleneck). Error bars: ± SD. Functions ranked by mean decrease. AUC, the area under the threshold curve.

**
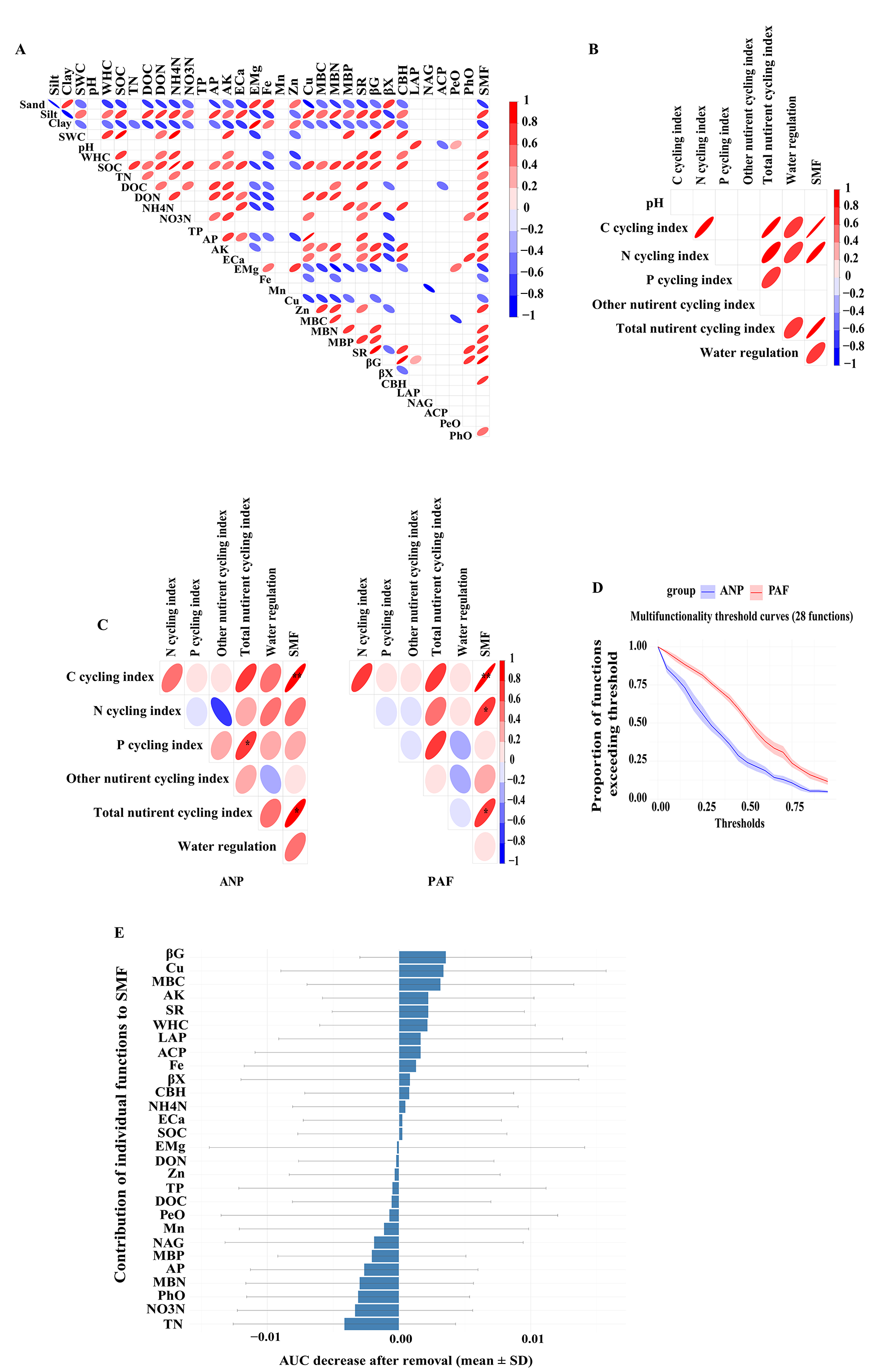
**

**Figure S4. Changes in soil microbial** **α-diversity as affected by different plantations in a subtropical karst area.** (A-D) Soil bacterial α-diversity; (E-H) Soil fungal α-diversity; (I-L) Soil protistan α-diversity. ANP, *Alnus nepalensis* plantations; PAF, *Pinus armandii Franch* plantations.


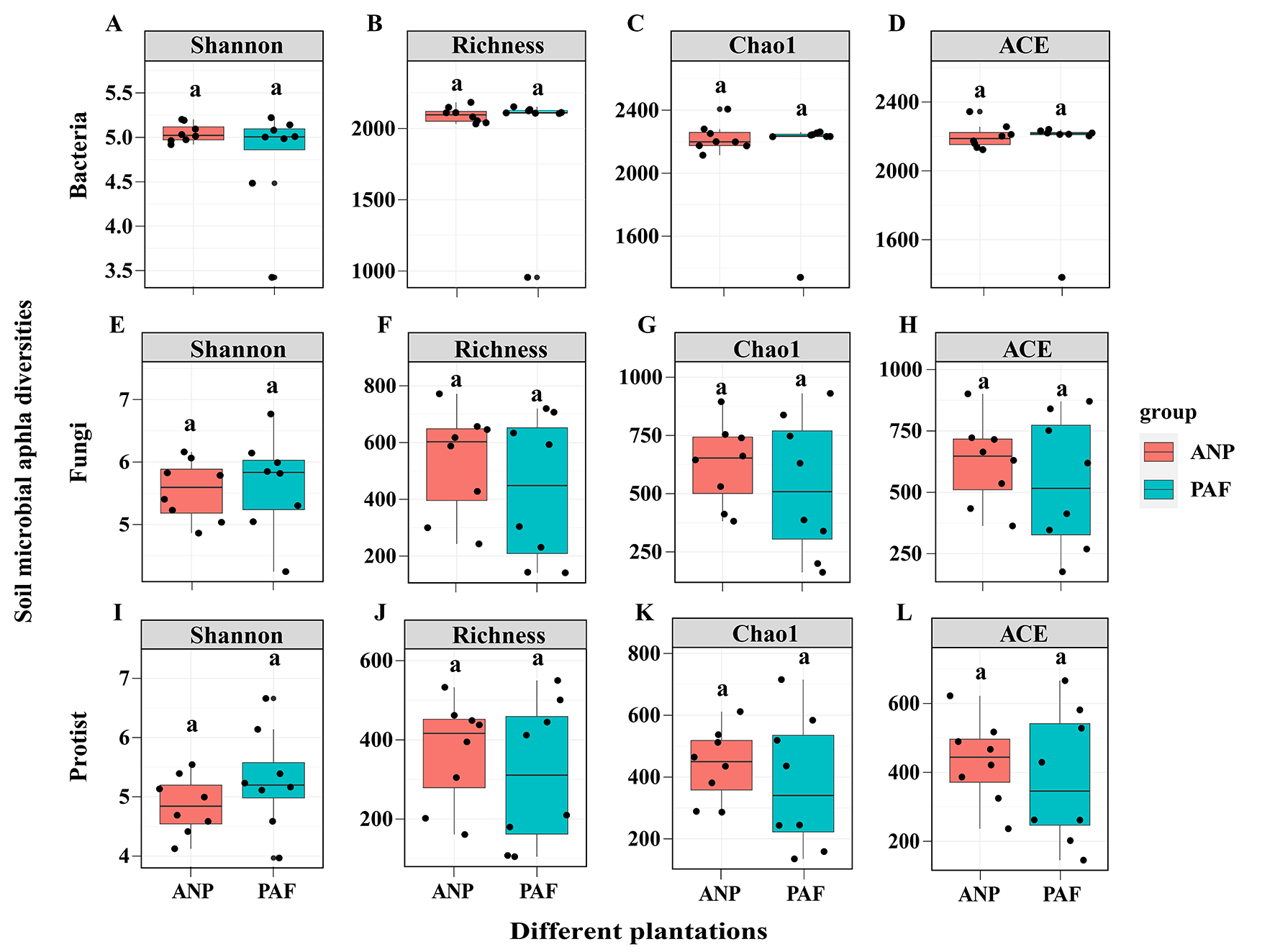


**Figure S5.** **The relationship between soil bacterial (A-D), fungal (E-H), and protistan (I-L) α-diversity and** **SMF.** ANP, *Alnus nepalensis* plantations; PAF, *Pinus armandii Franch* plantations.


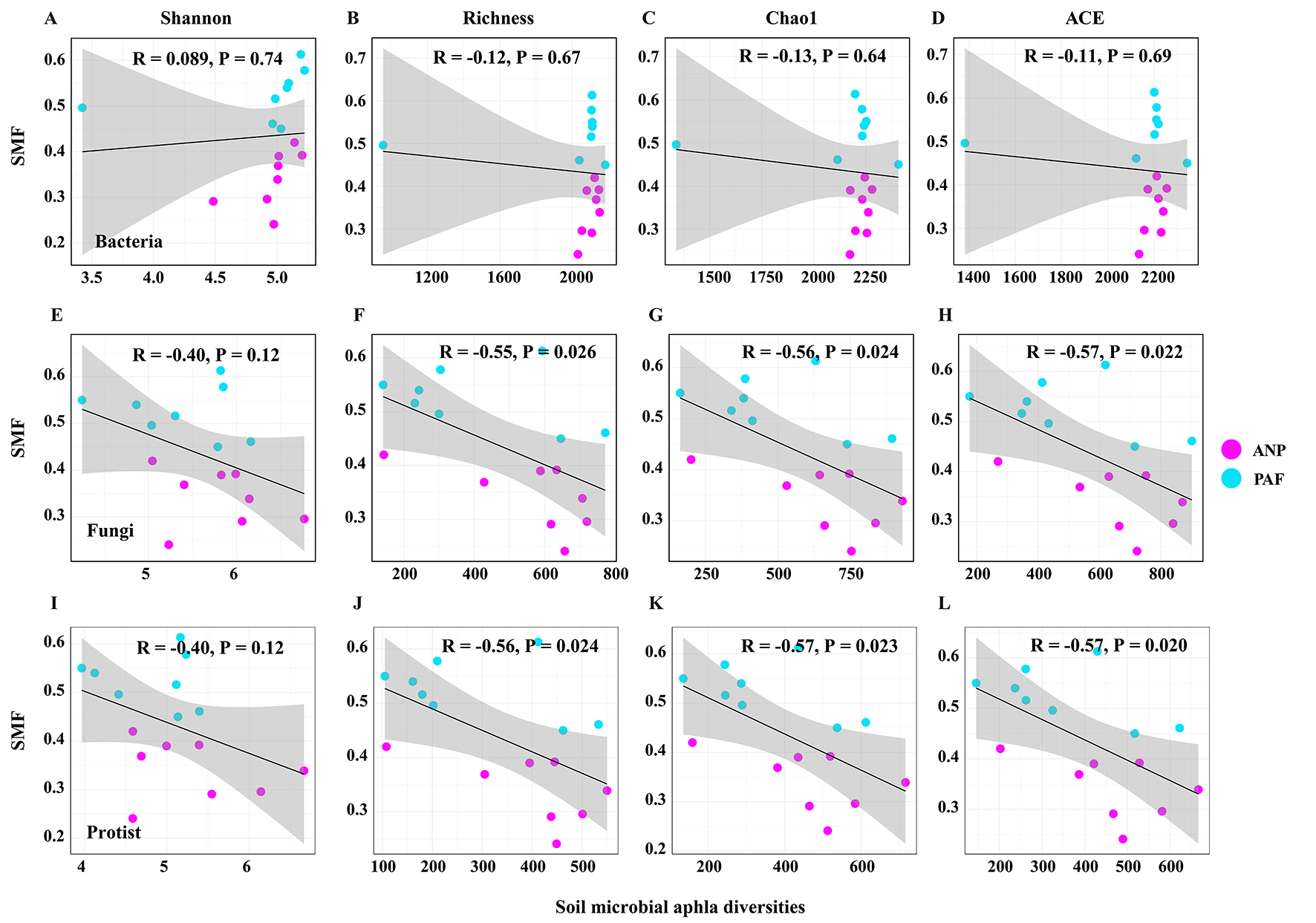


**Figure S6.** **The alteration in bacterial (A), fungal (B), and (C) protistan community composition** **was visualized by using NMDS analysis in different types of karst plantation.** ANP, *Alnus nepalensis* plantations; PAF, *Pinus armandii Franch* plantations.

**
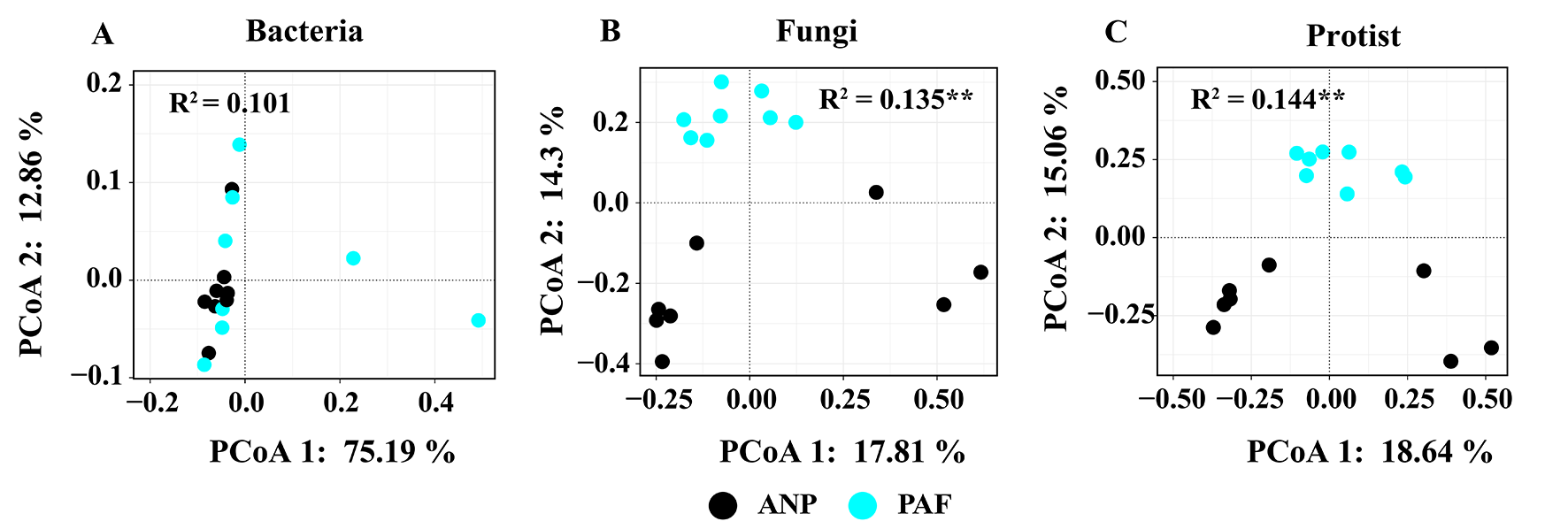
**

**Figure S7.** **(A) Effect of different karst plantations on the cumulative relative abundance of sASV in different functional modules.** **(B) Kingdom-level changes in bacterial, fungal, and protistan species across modules were analyzed using sASVs. (C) The cumulative relative abundance of sASV in each module in the two different plantations in the subtropical karst region. (D) The relationships between Modules (1 and 2) and SMF in different karst plantations are shown. Black lines indicate ordinary least squares regressions, with shaded areas representing the 95% confidence interval.** ANP, *Alnus nepalensis* plantations; PAF, *Pinus armandii Franch* plantations.


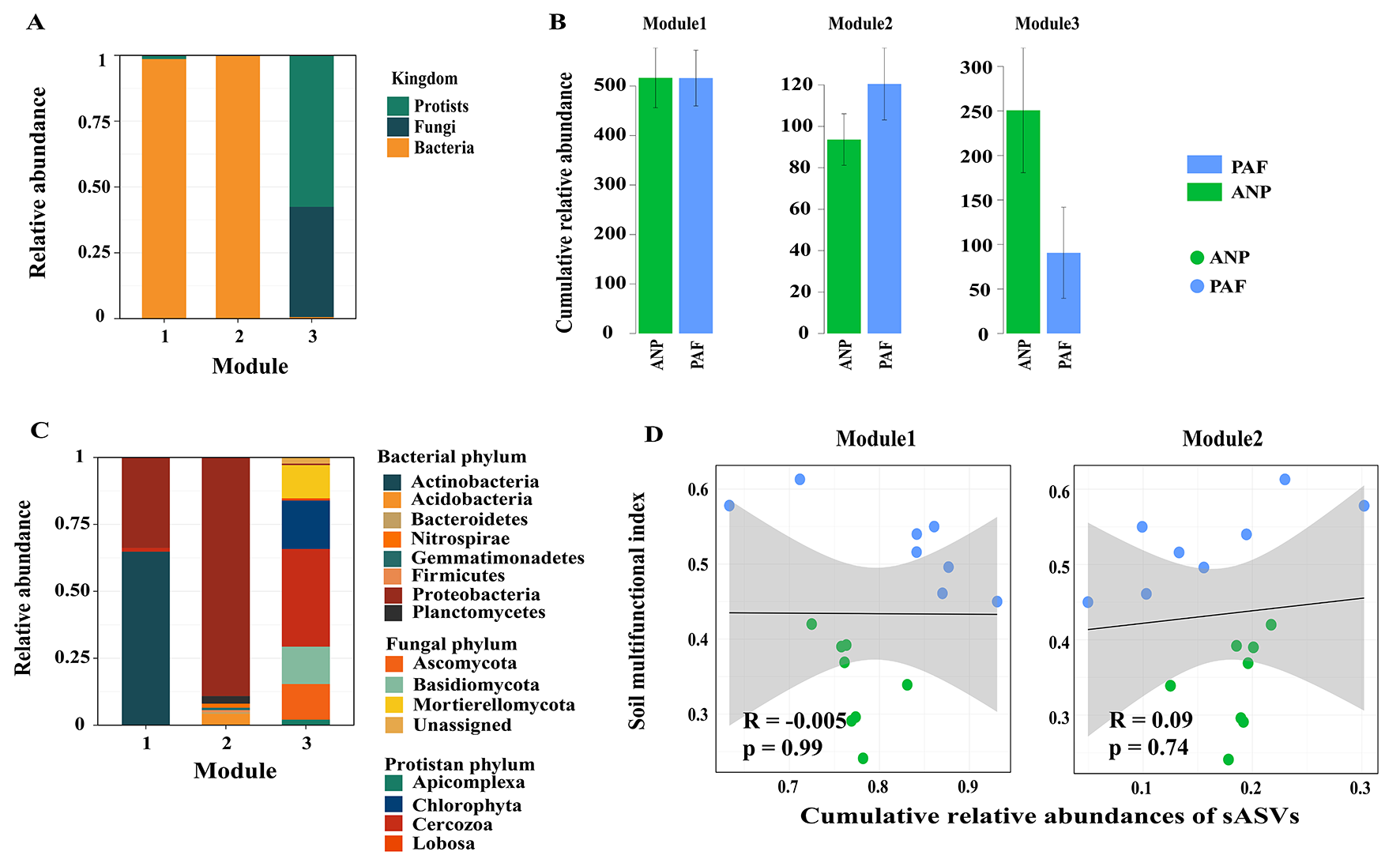


**Figure S8. Spearman correlation coefficients among** **dominant taxa of bacterial, fungal and protistan communities in module 3 at the genus level and SMF.** The blue and red ellipses indicate positive or negative relationships among the variables, respectively. The absence of ellipse filling indicates no significant correlation.


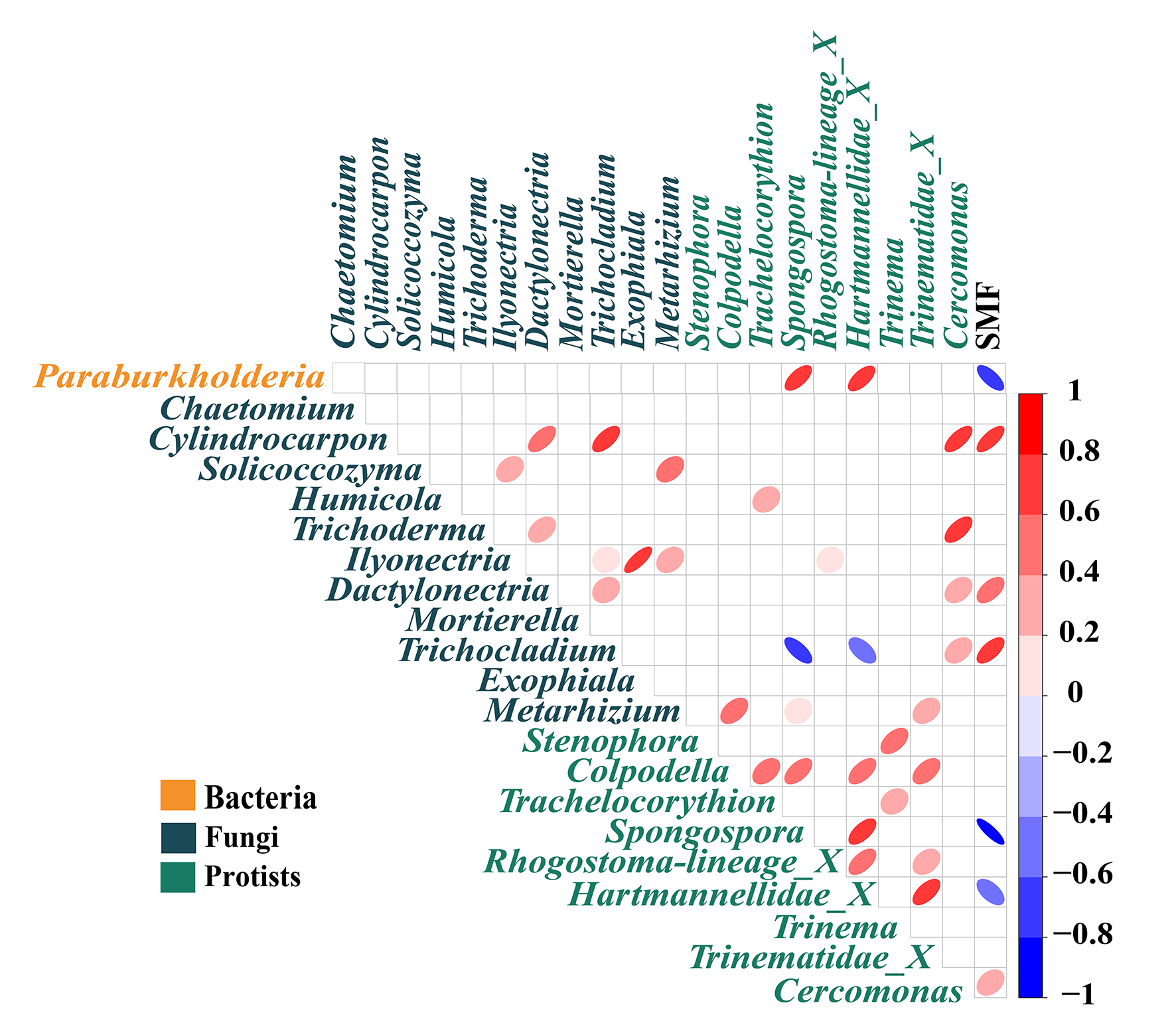


**Figure S9.** **Relationships between the relative abundances of dominant taxa of the bacterial (A), fungal (B-D) and protistan (E-G) communities in module 3 and SMF.** The black lines are fitted by ordinary least squares regressions. Shaded areas are 95% confidence intervals for the fitting. ANP, *Alnus nepalensis* plantations; PAF, *Pinus armandii Franch* plantations.


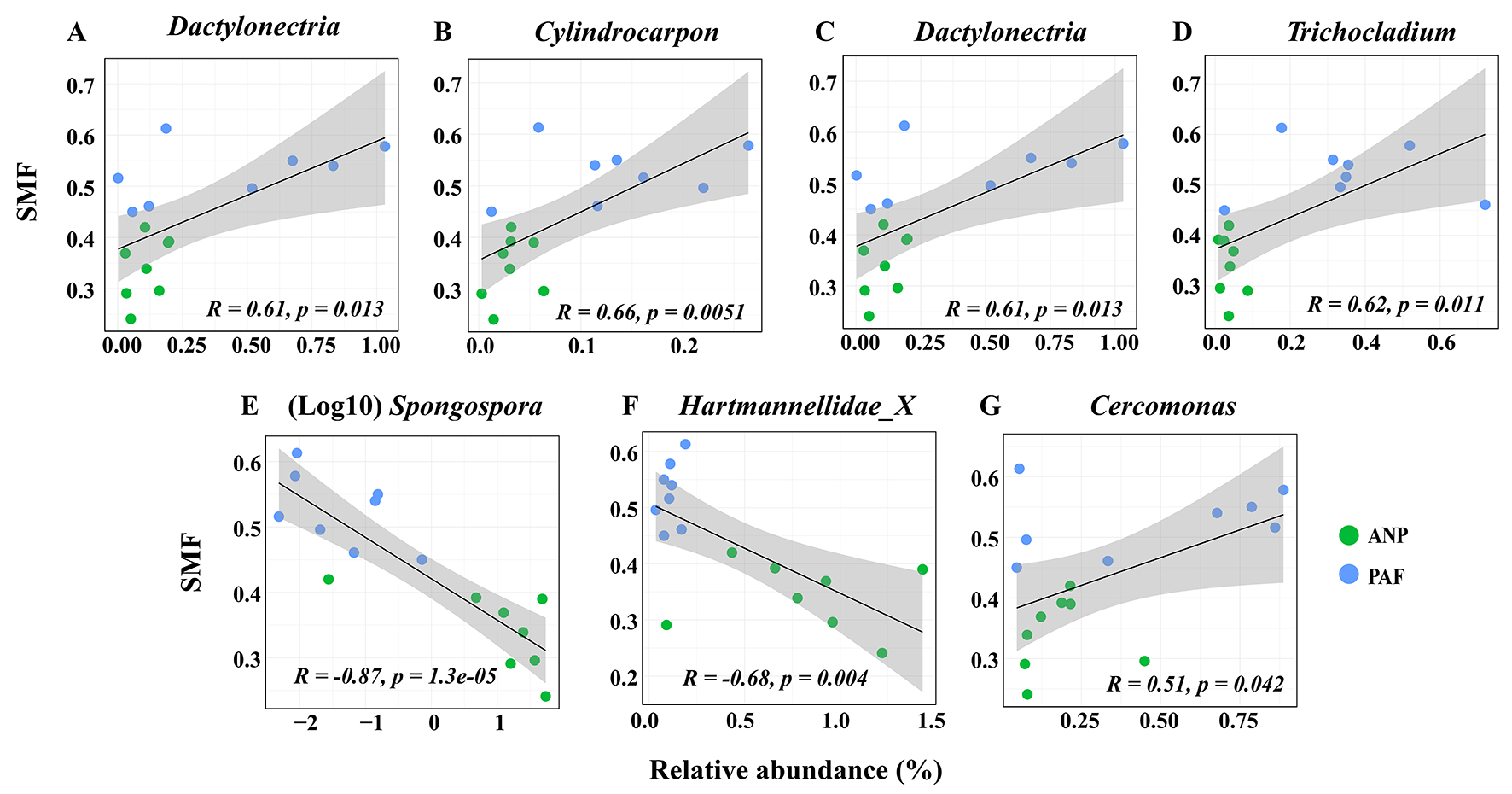


**Figure S10.** **Heatmap indicating** **the differences between the dominant taxa in module 3 of the microbial co-occurrence network under different plantations in subtropical karst areas.** ANP, *Alnus nepalensis* plantations; PAF, *Pinus armandii Franch* plantations.


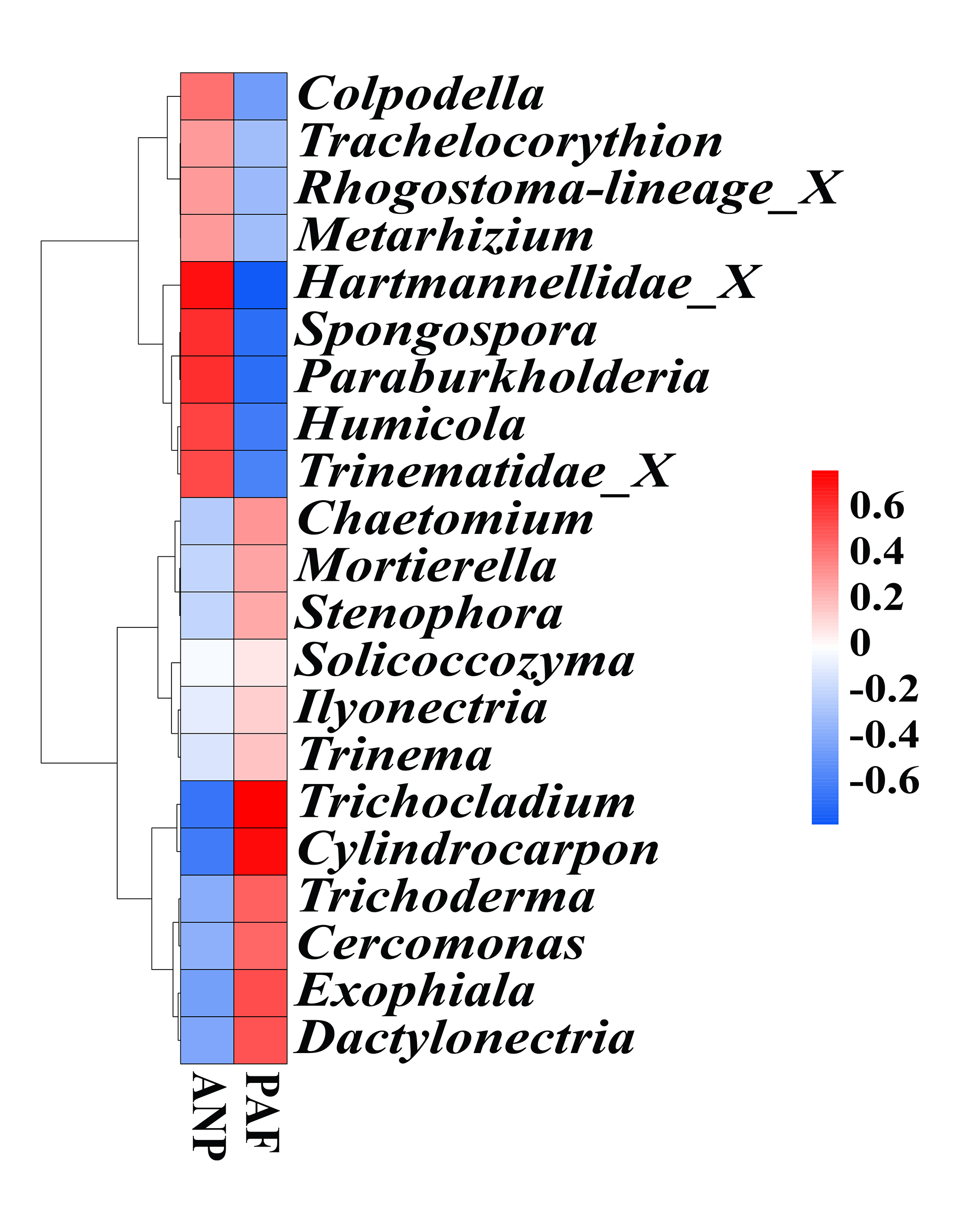


**References**

Delgado-Baquerizo, M., A. M. Oliverio, T. E. Brewer, A. Benavent-González, D. J. Eldridge, R. D. Bardgett, F. T. Maestre, B. K. Singh, and N. Fierer. 2018. A global atlas of the dominant bacteria found in soil. Science **359**:320-325.

Deng, Y., Y.-H. Jiang, Y. Yang, Z. He, F. Luo, and J. Zhou. 2012. Molecular ecological network analyses. BMC Bioinformatics **13**:113.

Duan, C., X. Li, C. Li, P. Yang, Y. Chai, and W. Xu. 2023. Positive effects of fungal β diversity on soil multifunctionality mediated by pH in the natural restoration succession stages of alpine meadow patches. Ecological Indicators **148**:110122.

Gao, M., C. Xiong, C. Gao, C. K. M. Tsui, M.-M. Wang, X. Zhou, A.-M. Zhang, and L. Cai. 2021. Disease-induced changes in plant microbiome assembly and functional adaptation. Microbiome **9**:187.

Kang, Y., L. Shen, C. Li, Y. Huang, and L. Chen. 2024. Effects of vegetation degradation on soil microbial communities and ecosystem multifunctionality in a karst region, southwest China. Journal of Environmental Management **363**:121395.

Li, J., L. Yang, M. Fan, and Z. Shangguan. 2022. Plantation vegetation restoration enhances the relationship between rhizosphere microbial diversity and soil multifunctionality. Land Degradation & Development **33**:3630-3640.

Li, Y., X. Han, B. Li, Y. Li, X. Du, Y. Sun, Q. Li, and T. Martijn Bezemer. 2023. Soil addition improves multifunctionality of degraded grasslands through increasing fungal richness and network complexity. Geoderma **437**:116607.

Ma, B., H. Wang, M. Dsouza, J. Lou, Y. He, Z. Dai, P. C. Brookes, J. Xu, and J. A. Gilbert. 2016. Geographic patterns of co-occurrence network topological features for soil microbiota at continental scale in eastern China. The ISME Journal **10**:1891-1901.

Maestre, F. T., J. L. Quero, N. J. Gotelli, A. Escudero, V. Ochoa, M. Delgado-Baquerizo, M. García-Gómez, M. A. Bowker, S. Soliveres, C. Escolar, P. García-Palacios, M. Berdugo, E. Valencia, B. Gozalo, A. Gallardo, L. Aguilera, T. Arredondo, J. Blones, B. Boeken, D. Bran, A. A. Conceição, O. Cabrera, M. Chaieb, M. Derak, D. J. Eldridge, C. I. Espinosa, A. Florentino, J. Gaitán, M. G. Gatica, W. Ghiloufi, S. Gómez-González, J. R. Gutiérrez, R. M. Hernández, X. Huang, E. Huber-Sannwald, M. Jankju, M. Miriti, J. Monerris, R. L. Mau, E. Morici, K. Naseri, A. Ospina, V. Polo, A. Prina, E. Pucheta, D. A. Ramírez-Collantes, R. Romão, M. Tighe, C. Torres-Díaz, J. Val, J. P. Veiga, D. Wang, and E. Zaady. 2012. Plant Species Richness and Ecosystem Multifunctionality in Global Drylands. Science **335**:214-218.

Xiong, C., Y.-G. Zhu, J.-T. Wang, B. Singh, L.-L. Han, J.-P. Shen, P.-P. Li, G.-B. Wang, C.-F. Wu, A.-H. Ge, L.-M. Zhang, and J.-Z. He. 2021. Host selection shapes crop microbiome assembly and network complexity. New Phytologist **229**:1091-1104.
